# Solution structure of multi-domain protein ER-60 studied by aggregation-free SAXS and coarse-grained-MD simulation

**DOI:** 10.1038/s41598-021-85219-0

**Published:** 2021-03-11

**Authors:** Aya Okuda, Masahiro Shimizu, Ken Morishima, Rintaro Inoue, Nobuhiro Sato, Reiko Urade, Masaaki Sugiyama

**Affiliations:** Institute for Integrative Radiation and Nuclear Science, Kyoto University, Kumatori, Sennan-gun, Osaka 590-0494 Japan

**Keywords:** Structural biology, SAXS

## Abstract

Multi-domain proteins (MDPs) show a variety of domain conformations under physiological conditions, regulating their functions through such conformational changes. One of the typical MDPs, ER-60 which is a protein folding enzyme, has a U-shape with four domains and is thought to have different domain conformations in solution depending on the redox state at the active centres of the edge domains. In this work, an aggregation-free small-angle X-ray scattering revealed that the structures of oxidized and reduced ER-60 in solution are different from each other and are also different from those in the crystal. Furthermore, structural modelling with coarse-grained molecular dynamics simulation indicated that the distance between the two edge domains of oxidized ER-60 is longer than that of reduced ER-60. In addition, one of the edge domains has a more flexible conformation than the other.

## Introduction

Multi-domain proteins (MDPs) constitute one of the major categories of ubiquitous proteins, presumably accounting for over half of all human proteins^1,2^. MDPs can have several domain conformations and dynamically transit between them under physiological conditions, in solution. The domain conformation plays a role in the association with other proteins, nucleic acids, small molecules, etc., therefore determining a specific MDP function. Thus, elucidating the domain conformations and their dynamics in solution is important.

We have focused on ER-60, also called ERp57, GRP58, or PDIA3^3–13^, which is a protein folding enzyme belonging to the protein disulphide isomerase family. ER-60 is also a typical MDP consisting of four thioredoxin-like domains, **a**, **b**, **b′**, and **a′**^14,15^. Two edge domains, **a** and **a′**, have catalytically active Cys–Gly–His–Cys (CGHC) motifs, which interconvert between oxidized (disulphide) and reduced (dithiol) forms. The oxidized form catalyses the oxidation reaction of dithiol on a substrate (an unfolded protein), whereas the reduced form catalyses the reduction or isomerization of disulphide bonds on a substrate. In this catalytic process, ER-60 must have appropriate domain conformations to perform the formation and rearrangement of disulphide bonds on the substrate^16^. Therefore, it is assumed that ER-60 can change its domain conformation according to the redox state of the CGHC motif to either oxidize or reduce the substrate.

The atomic-scale structure of full-length human ER-60 has only been revealed in complex with tapasin in an X-ray crystallographic study^18^. As shown in Supplementary Fig. S1a, in the complex, the four domains of ER-60 are arranged in a twisted U-shape and the N-terminal domain of tapasin is sandwiched between the two edge domains, **a** and **a′**. This suggested that, under physiological conditions, namely in solution, the domains of ER-60 could also form a similar U-shape and associate with a substrate using the **a** and **a′** domains, and that the conformation of the **a** and **a′** domains could be changed according to the redox state of the CGHC motif. To confirm this, the structure of ER-60 in solution, especially the conformation of the four domains, and its dependence on the redox state of the CGHC motif, must be revealed.

For this purpose, small-angle X-ray scattering (SAXS) is a powerful tool that can be used to observe the structure of biomacromolecules in solution. However, SAXS is sensitive to unfavourable contaminations and aggregates in solution, especially large molecules when even in small amounts^18–21^. Therefore, we carefully prepared highly purified oxidized and reduced ER-60, examined the quality of the samples, and conducted state-of-the-art SAXS methods combined with size-exclusion chromatography (SEC) (SEC-SAXS)^22^ and with analytical ultracentrifugation (AUC) (AUC-SAS)^21^. The integrative organization of these methods allowed us to achieve high-precision aggregation-free SAXS profiles.

To visually understand domain conformation, we built three-dimensional structures of oxidized and reduced ER-60 in solution by computational modelling using the SAXS data. Prior to the computational modelling, we compared two crystal structures of the **b**–**b′** domains: one is the **b**–**b′** domains of full-length ER-60 in the tapasin complex (3F8U) and the other is only **b**–**b′** domains, ER-60 truncated the **a** and **a′** domains^23^ (2H8L) (Supplementary Fig. S1b). Since they were almost the same as indicated by the calculated root mean square deviation of *ca.* 0.5 (Supplementary Fig. S1b), we assumed that the conformation of the **b** and **b′** domains are almost fixed, while that of the two edge domains, **a** and **a′**, can be flexible. Under this assumption, we designed the proper interaction potential of intra- and inter-domains and then performed coarse-grained molecular dynamics (CG-MD) simulations to search for the possible structural space of ER-60. Compared to standard atomistic MD simulations, the CG-MD approach drastically accelerates structural space sampling^24,25^. The sampled models were screened with the aggregation-free SAXS profiles of the oxidized and reduced ER-60 forms.

Finally, we succeeded in deriving two sets of structural models from simulation snapshots, which represented the solution structures of oxidized and reduced ER-60, respectively. From the structural models, we examined the differences in their domain conformations.

## Results and discussion

### Solution scattering of ER-60

**Figure 1:**
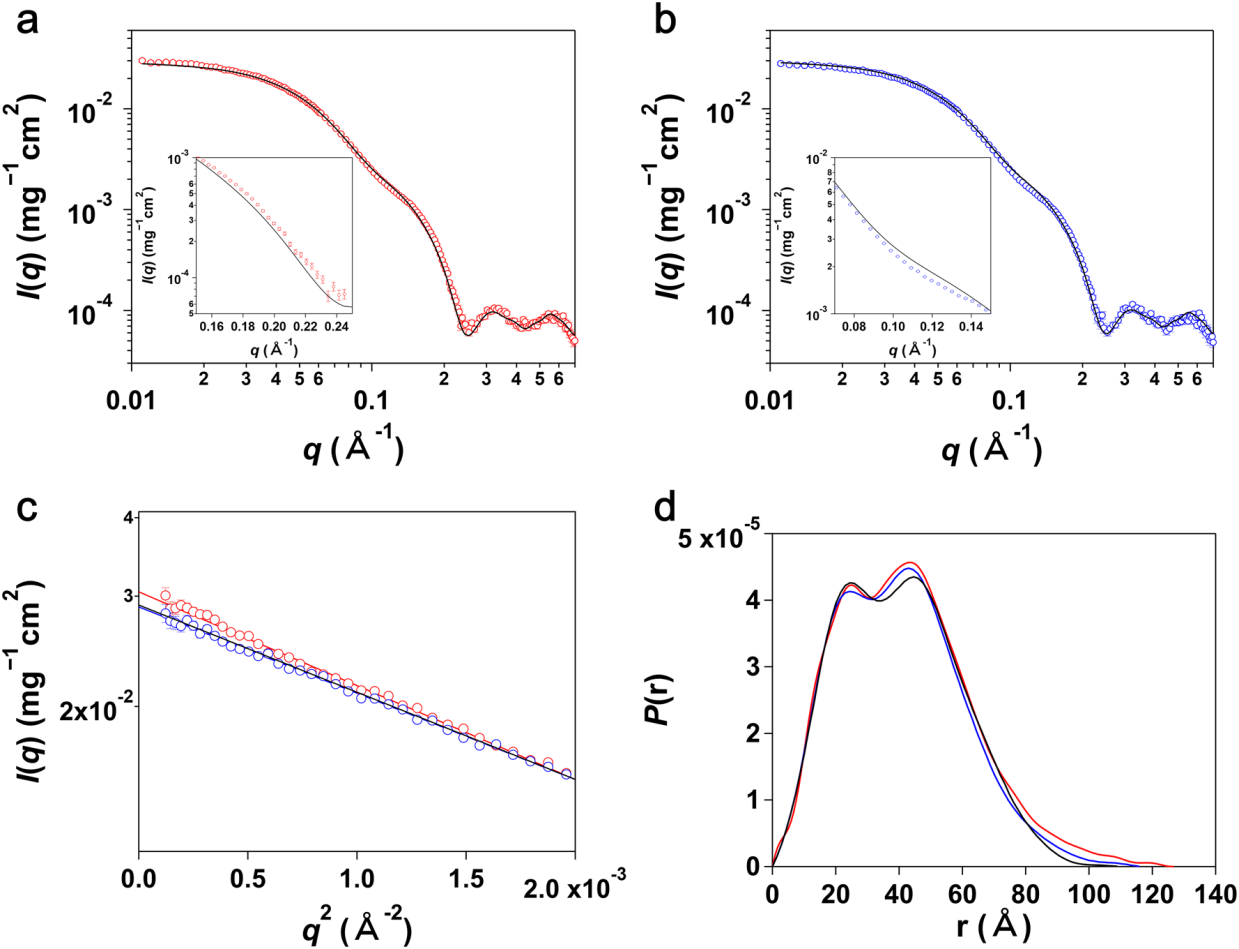
Solution scattering of ER-60. (**a**, **b**) Small-angle X-ray scattering (SAXS) profiles of oxidized (**a**, red circles) and reduced (**b**, blue circles) ER-60, respectively. Black lines indicate the profiles calculated from the crystal structure (3F8U). Inset panels are expanded views of them in a *q* range of 0.17–0.25 Å^−1^ (**a**) and 0.07–0.15 Å^−1^ (**b**), respectively. (**c**) Guinier plots of oxidized (red circles) and reduced (blue circles) ER-60. The red and blue straight lines indicate the results of the least square fitting using the Guinier formula: *I*(*q*) = *I*(0)exp(− (*R*g/3)^2^*q*^2^), where *R*_g_ is the gyration radius and *I*(0) is the forward scattering intensity. The black line indicates the profiles of the crystal structure (3F8U) in the Guinier plot. (**d**) Pair distance distribution function, *P*(*r*), of oxidized (red line) and reduced (blue line) ER-60 calculated using GNOM software^36^. The black line indicates the *P*(*r*) of the crystal structure (3F8U).

Figure 1a,b show the aggregation-free SAXS profiles of oxidized and reduced ER-60, respectively, which were obtained by the integrative organization of the methods described in the “Aggregation-free SAXS data” subsection of the “Methods” section. First, we compared the observed SAXS profiles of ER-60 with that calculated using the crystal structure (3F8U). As emphasized in the inset panels of Fig. 1a,b, the SAXS profiles of oxidized and reduced ER-60 in solution were different from that calculated using the crystal structure. Furthermore, the calculated values of χ^2^ between the observed SAXS profiles and that calculated using the crystal structure were 13.5 and 10.7 for oxidized and reduced ER-60, respectively. This means that the complex-free structures in solution are different from the crystal structure of the complexed protein.

Next, we examined the structural differences between oxidized and reduced ER-60. Their Guinier plots and distance distribution functions are displayed in Fig. 1c,d, respectively. The gyration radii *R*_g_ with Guinier analyses and the longest distance *D*_max_ from the distance distribution functions are presented in Table 1. In addition, the elution volumes for oxidized and reduced ER-60 on SEC are also described in Table 1. The elution charts are shown in Supplementary Fig. S2. The average mass values of the two samples, oxidized and reduced ER-60, were measured using LC-ESI-TOF mass spectrometry. The measured average mass values were the same as those calculated from their amino acid sequences (Table 1 and Supplementary Fig. S3). These results and SDS-PAGE of the samples (Supplementary Fig. S4d,e) showed that the oxidized and reduced samples were intact without any truncation or degradation. Therefore, the larger *R*_g_, the longer *D*_max_, and the smaller elution volume of the oxidized ER-60 compared to reduced ER-60 clearly indicated that oxidized ER-60 forms a more expanded structure than reduced ER-60 in solution.

**Table 1:** The values obtained from the SAXS measurement, SEC, and ESI-TOF MS of ER-60.

	*R*_g_ (Å)	*D*_max_ (Å)	Measured average mass (Da)	Calculated average mass (Da)	Peak top of elution volume (mL)
**ER-60**					
Oxidized	32.1 ± 0.2	126.7 ± 4.9	54,259.3	54,260.5	13.48
Reduced	30.8 ± 0.2	117.1 ± 4.2	54,264.0	54,264.6	13.60
Crystal	31.0	109.0	–	52,807.8	–

In summary, in terms of χ^2^, there are no large differences between the three structures: the oxidized and reduced ER-60, and the crystal structures of the complexed protein. However, we have succeeded to find the small but certain differences with our careful sample preparation and high-precision SAXS measurements.

### Structural modelling of ER-60

We built structural and dynamical models for both forms, which reflect their differences. Considering the structural differences between two forms and the crystal form are not large, it is assumed that the solution structures for both forms could distribute homogeneously around the crystal structure. Therefore, we extensively sampled structures with various domain conformations by CG-MD. Subsequently, the structures that reproduced the experimental SAXS profiles for both forms were collected from the sampled structures.

**Figure 2:**
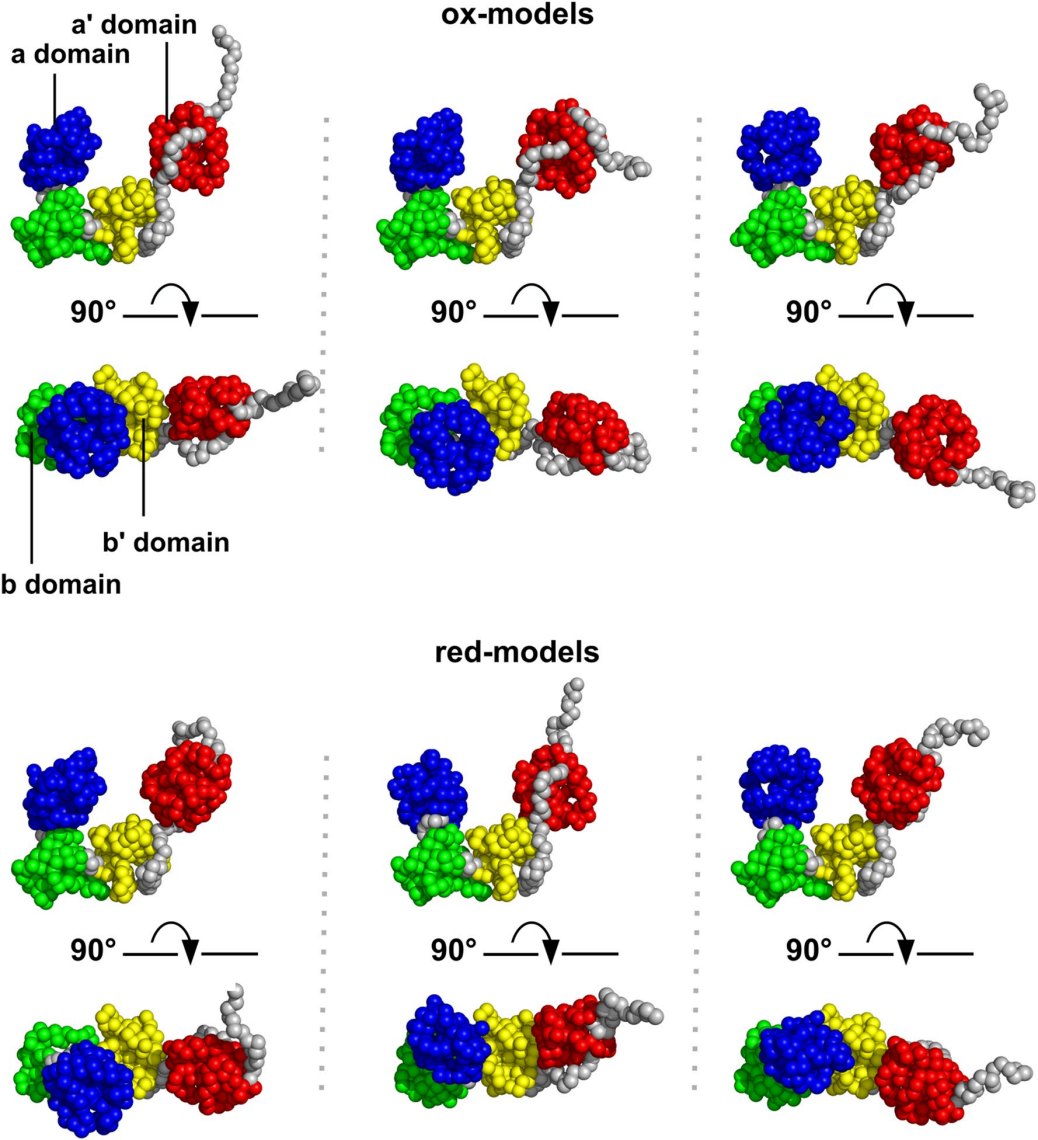
Oxidized ER-60 (ox-models) and reduced ER-60 (red-models) models. A total of six models were taken for oxidized and reduced ER-60 (three each) from the extracted ox- and red-models and are shown from two viewpoints. Here, the **a**, **b**, **b′**, **a′** domains and the inter-domain regions are coloured in blue, green, yellow, red, and gray, respectively.

First, we took 800,000 snapshots with CG-MD (see the details in the “Supplemental methods”). Then, SAXS profiles were calculated for all snapshots. The structural models reproducing the observed SAXS profile of oxidized ER-60 (ox-models) were extracted from all snapshots using two evaluation criteria: χ^2^ < 7.0 and 31.9 Å < *R*_g_ < 32.3 Å. The total number of ox-models was 230. The structural models reproducing the observed SAXS profile of reduced ER-60 (red-models) were extracted with two evaluation criteria: χ^2^ < 7.0 and 30.6 Å < *R*_g_ < 31.0 Å. The total number of red-models was 1572. Figure 2 shows typical extracted structures, indicating that all ox- and red-models have a twisted U-shape.

**Figure 3:**
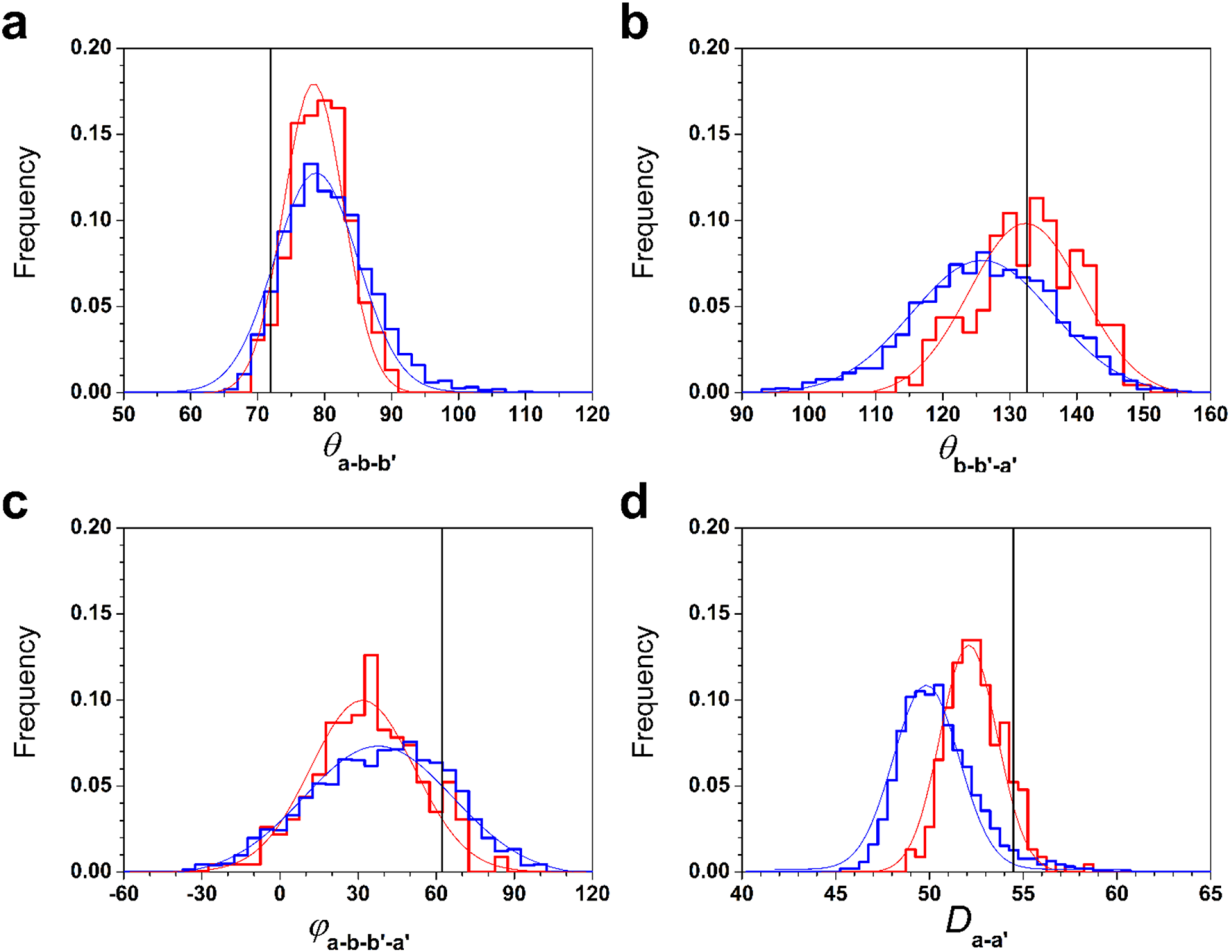
Structural analysis of the ER-60 models. Distributions of *θ*_**a–b–b′**_ (**a**), *θ*_**b–b′–a′**_ (**b**), and *φ*_**a–b–b′–a′**_ (**c**), and the *D*_**a–a′**_ (**d**) are shown with red and blue histograms. The frequency is the fraction of the count in each bin divided by the total number of models. The values of the crystal structure are also shown by black lines. Histograms were fit by Gaussians for visibility. The eye-guide curves are shown with red and blue lines for ox- and red-models, respectively.

Subsequently, we investigated the differences in domain conformation between the ox- and red-models. Three angles and one distance, which characterise domain conformation, were calculated for the ox- and red-models. As shown in Supplementary Fig. S5, *θ*_**a**–**b**–**b′**_ and *θ*_**b**–**b′**–**a′**_ are defined as angles formed by the **a**, **b**, **b′** and **b**, **b′**, **a′** domains, respectively, while *φ*_**a**–**b**–**b′**–**a′**_ is defined as the dihedral angle formed by all four domains and *D*_**a**–**a′**_ is the distance between the centre of mass (COM) of the **a** and **a′** domains. As shown in Fig. 3a,b, *θ*_**a**–**b**–**b′**_ showed similar ranges distributed around 75º in both models, whereas *θ*_**b**–**b′**–**a′**_ showed relatively different ranges, which were 110°–155° and 95°–150° for ox- and red-models, respectively. This suggested that the **a** domains of the ox- and red-models have almost the same position, while the **a′** domains of the ox-models locate at a more open position than those of the red models. Moreover, as shown in Fig. 3c, *φ*_**a–b–b′–a′**_ had a similar range distribution in both models, meaning that the degrees of twisting of the U-shape were almost the same. The effect of the more open conformation in the ox-models is clearly recognized in the *D*_**a**–**a′**_. As shown in Fig. 3d, the *D*_**a**–**a′**_ of the ox-models was distributed over a longer range than that of the red models.

**Figure 4:**
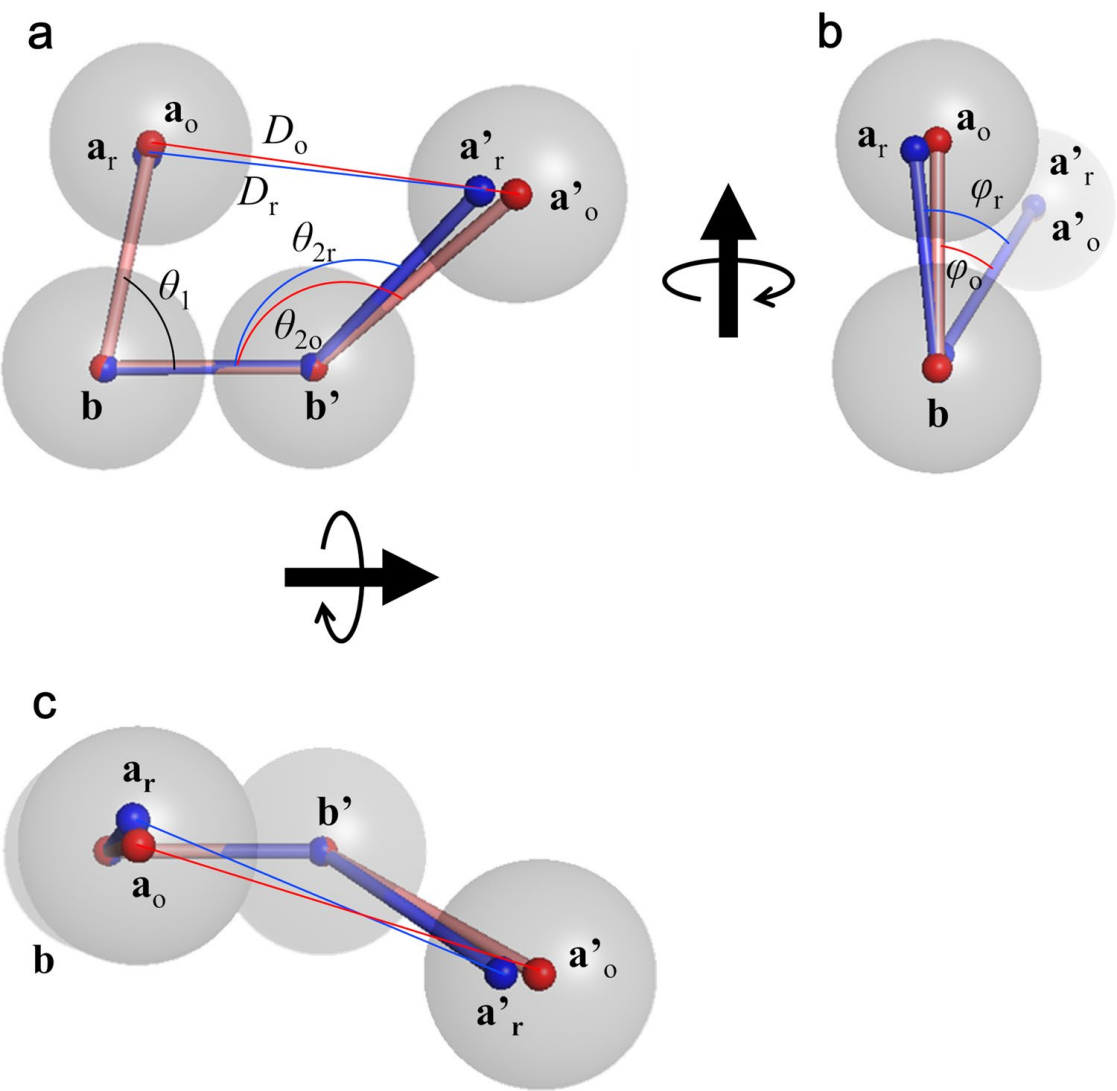
Ball-and-stick models of ER-60. The averaged position of each domain is shown with red ball-and-stick models and with blue ball-and-stick models for ox- and red-models, respectively. The ox- and red-models are aligned by the **b** and **b′** domains. Gray balls roughly indicate domains as an eye guide. *θ*_1_ is the *θ*_**a–b–b′**_. *θ*_2o_ and *θ*_2r_ indicate the *θ*_**b–b′–a′**_ of the ox- and red-models, respectively. *φ*_o_, and *φ*_r_ indicate the *φ*_**a–b–b′–a′**_ of the ox- and red-models, respectively.

The structural images are shown in Fig. 4. For visualisation, the **b** and **b′** domains of all models were superimposed, and then the positions of the COM of every domain were averaged over the ox- and red-models, respectively. The COM-positions were expressed with small spheres and the subscripts for **a** and **a′** domains, o and r, corresponding to ox- and red-model(s), respectively. As shown in Fig. 4, **a**_o_ and **a**_r_ are almost the same, but **a′**_o_ is slightly shifted to be more open than **a′**_r_. The distance between averaged **a**_o_ position and averaged **a**_r_ position was 3.4 Å, whereas that between averaged **a′**_o_ and averaged **a′**_r_ was 4.7 Å. This also suggests that the structures of oxidized and reduced ER-60 mainly differ in the position of the **a′** domain. The results are summarized in Table 2.

**Table 2:** The values from the structural analysis of the ER-60 models.

	*θ*_**a–b–b′**_ [°]	*θ*_**b–b′–a′**_ [°]	*φ*_**a–b–b′–a′**_ [°]	*D*_**a–a′**_ [Å]
Oxidized	78.4 ± 4.3	131.8 ± 7.6	31.4 ± 20.0	52.2 ± 1.5
Reduced	79.6 ± 6.4	125.4 ± 10.3	36.1 ± 25.8	50.3 ± 2.1
Crystal	72.0	132.6	62.2	54.5

**Figure 5:**
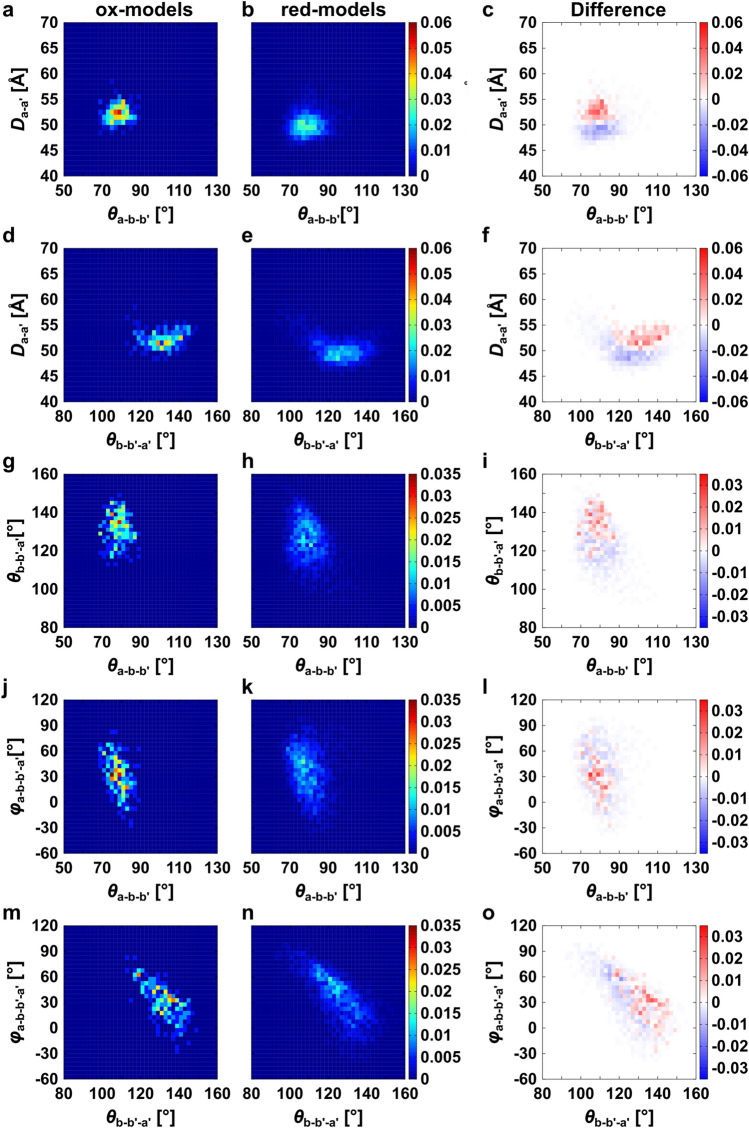
Two-dimensional mapping of the model structures around the **a** and **a′** domains. Distributions of correlations between two parameters, *θ*_**a–b–b′**_—*D*_**a–a′**_ (**a**, **b**), *θ*_**b–b′–a′**_—*D*_**a–a′**_ (**d**, **e**), *θ*_**a–b–b′**_—*θ*_**b–b′–a′**_ (**g**, **h**), *θ*_**a–b–b′**_—*φ*_**a–b–b′–a′**_ (**j**, **k**), and *θ*_**b–b′–a′**_—*φ*_**a–b–b′–a′**_ (**m**, **n**) of the ox- and red-models_._ Panels (**c**, **f**, **i**, **l**, **o**) show the subtraction of the frequency in the red-models from that in the ox-models.

**Figure 6:**
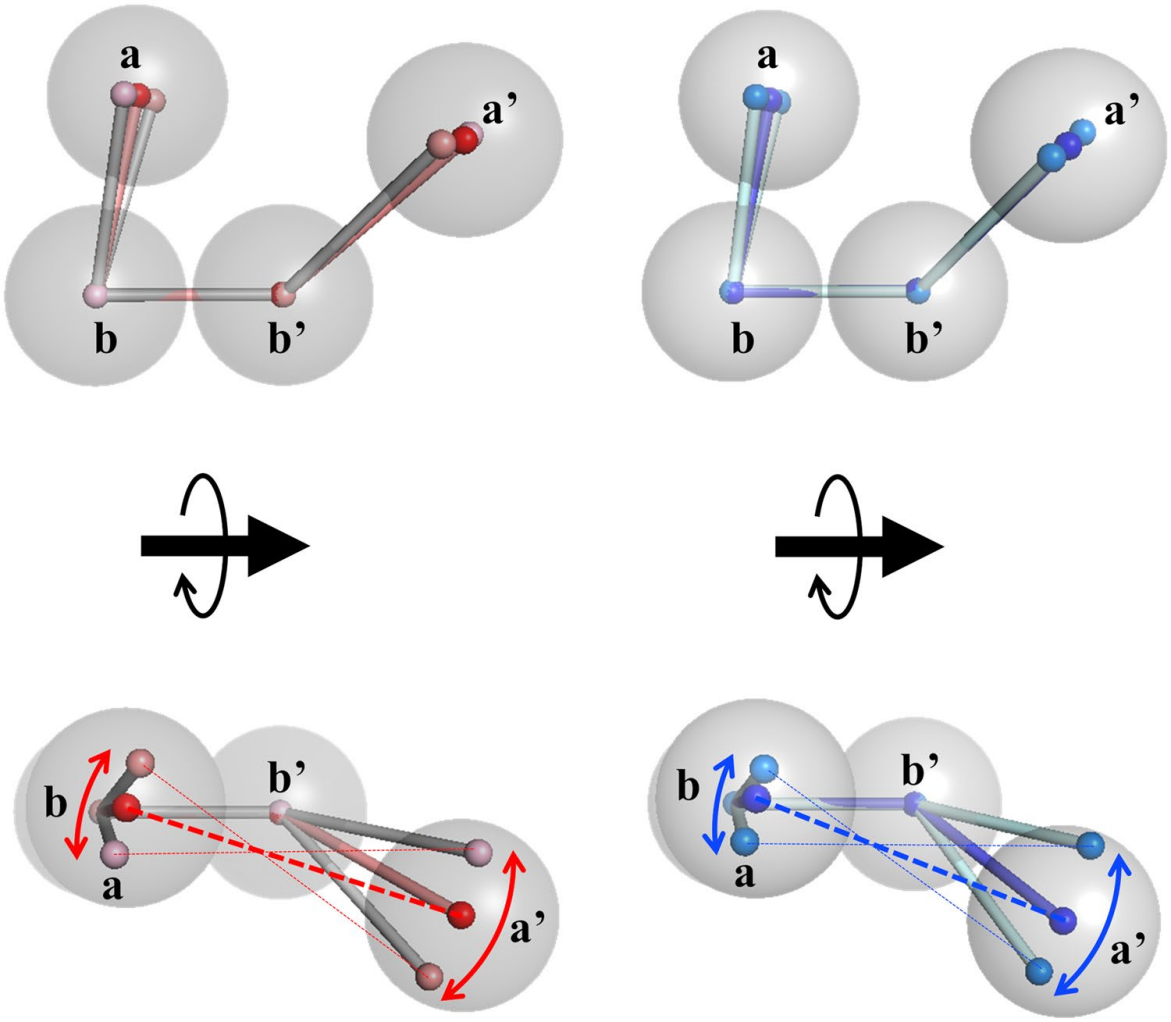
Conformational flexibility between the **a** and **a′** domains of ER-60. The conformational distribution of the domains is shown with red ball-and-stick models and with blue ball-and-stick models for ox- and red-models, respectively. The ox- and red-models are aligned by the **b** and **b′** domains. Gray balls roughly indicate domains as an eye guide. Coloured arrows indicate the expected ranges of flexibility in the **a** and **a′** domains.

The details of these conformational features were clarified by referring to the relations between the parameters. Figure 5 shows the contour maps of the frequency distribution as functions of two parameters in ox- and red-models and the difference between them. Here, we focused on the relations concerning the conformation of the **a′** domain. As shown in Fig. 5d,e, the **a′** domains in both the ox- and red-models were distributed keeping the distances *D*_**a**–**a′**_. This implied that the **a′** domain rotates around the **a** domain, which results in the twisting of the U-shape because the **a** domain is almost fixed. This motion is shown in Fig. 5m,n. In the case of the ox-models, when the **a′** domain shifts from *θ*_**b–b′–a′**_ = 140° to *θ*_**b–b′–a′**_ = 110°, meaning that the **a′** domain rises up to the **a** domain, the dihedral angle changes from *φ*_**a–b–b′–a′**_ = 0° to *φ*_**a–b–b′–a′**_ = 60°, meaning that the flat U-shape is being twisted (Fig. 5m). More correctly, the slightly fluctuated **a** domain (Δ*θ*_**a–b–b′**_ = 10°) correlated with the position shift of the **a′** domain (Δ*θ*_**b–b′–a′**_ = 30°). As shown in Fig. 5j,m, triple angle relations (*θ*_**a–b–b′**_, *θ*_**b–b′–a′**_, *φ*_**a–b–b′–a′**_) were 80°, 140°, 0° and 70°, 110°, 60°, respectively. The difference in conformational flexibility between the **a** and **a′** domains could be ascribed to the linkers between the **b** and **b′** domains. In the red-models, same conformational relations such as triple angle relations (*θ*_**a–b–b′**_, *θ*_**b–b′–a′**_, *φ*_**a–b–b′–a′**_) were observed from 80°, 140°, 0° to 70°, 110°, 60°. The difference from the ox-models is the shorter *D*_**a–a′**_ as described. These structural pictures are shown in Fig. 6. It should be noted that no correlations were found between the conformation of C-terminal loop of ER-60 and *θ*_**a–b–b′**_, *θ*_**b–b′–a′**_, *φ*_**a–b–b′–a′**_**,** or *D*_**a–a′**_ (Supplementary Fig. S6)**.**

**Figure 7:**
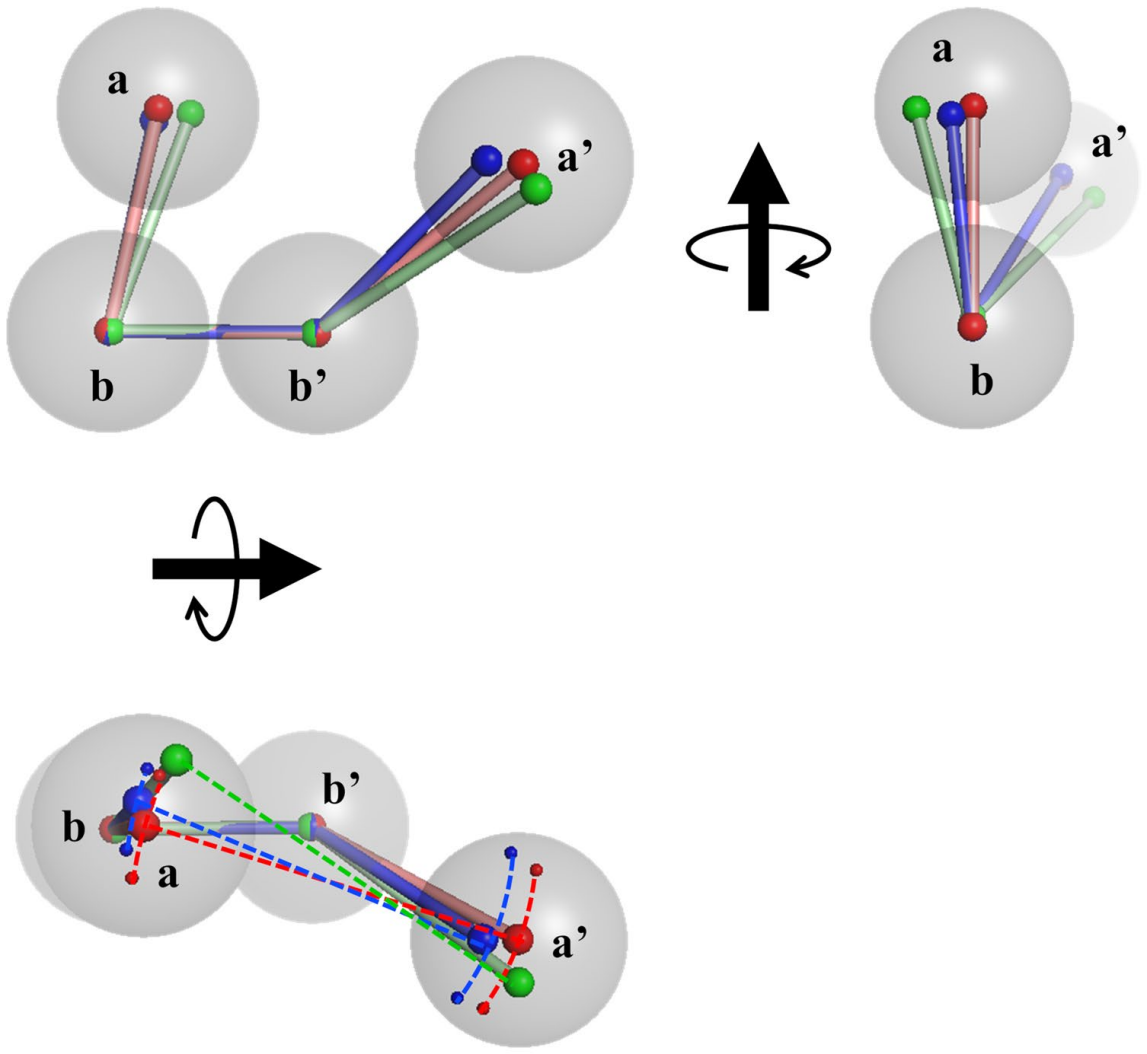
Comparison of the ball-and-stick models for ox- and red-models with that for the crystal structure. The averaged position of each domain is shown with red ball-and-stick models and with blue ball-and-stick models for ox- and red-models, respectively. The ox- and red-models and the crystal structure are aligned by the **b** and **b′** domains. The corresponding positions of the crystal structure are shown in the green ball-and-stick model. Gray balls roughly indicate domains as an eye guide. Dashed lines between smaller balls indicate the ranges of flexibility in the **a** and **a′** domains.

We also compared the ox- and red-models with the crystal structure. In Fig. 3a–d, the four angles of the crystal structure are indicated by black lines. Briefly, in the tapasin complex form, the **a** domain slightly turns to the **b** and **b′** domains, and the **a′** domain has the most open conformation and rotates by inserting tapasin between the **a** and **a′** domains, making their distance longer. As a result, the U-shape of the crystal structure is more twisted than those of the two complex-free structures, ox- and red-models, in solution. This is illustrated in Fig. 7. In addition, the conformational flexibility of the **a′** domain also contributes to the twisted structure in the crystal. In conclusion, complex-free ER-60 in solution acquires a less twisted structure than the complexed protein by releasing tapasin. The twisted form acquired by the conformational flexibility of the **a′** domain could contribute to capture a variety of proteins for the formation (oxidation) and cleavage (reduction) of disulphide bonds.

In summary, the combination of aggregation-free SAXS and CG-MD simulations is a powerful tool to analyse the structure of MDPs in solution, especially for domain conformation fluctuation.

Aggregation-free SAXS data of oxidized and reduced ER-60 were acquired by integrating the following procedures: highly purified sample preparation, step-by-step sample quality checks, SEC-SAXS, and AUC-SAS. The SAXS data indicated that both solution structures are different from the crystal structure of the tapasin-ER-60 complex and that the structure of oxidized and reduced ER-60 differs; the former is slightly more expanded than the latter. To analyse the structures and fluctuations of their domain conformations in more detail, three-dimensional structures were constructed with CG-MD simulation, which allows searching for a large phase space within a relatively short time compared with the all-atom MD simulation. CG-MD analysis revealed that the U-shape in the crystallographic structure is held in solution, but the distances between the **a** and **a′** domains are different (longer in the crystal, then in oxidized ER-60, and finally in reduced ER-60). Interestingly, the **a′** domain could fluctuate more than the **a** domain, may allow twisting of the U-shape. We speculate that the twisting resulting from the fluctuation of the **a′** domain may allow ER-60 to capture a variety of substrates. To probe this will be the next research target.

## Methods

### Aggregation-free SAXS

**Figure 8:**
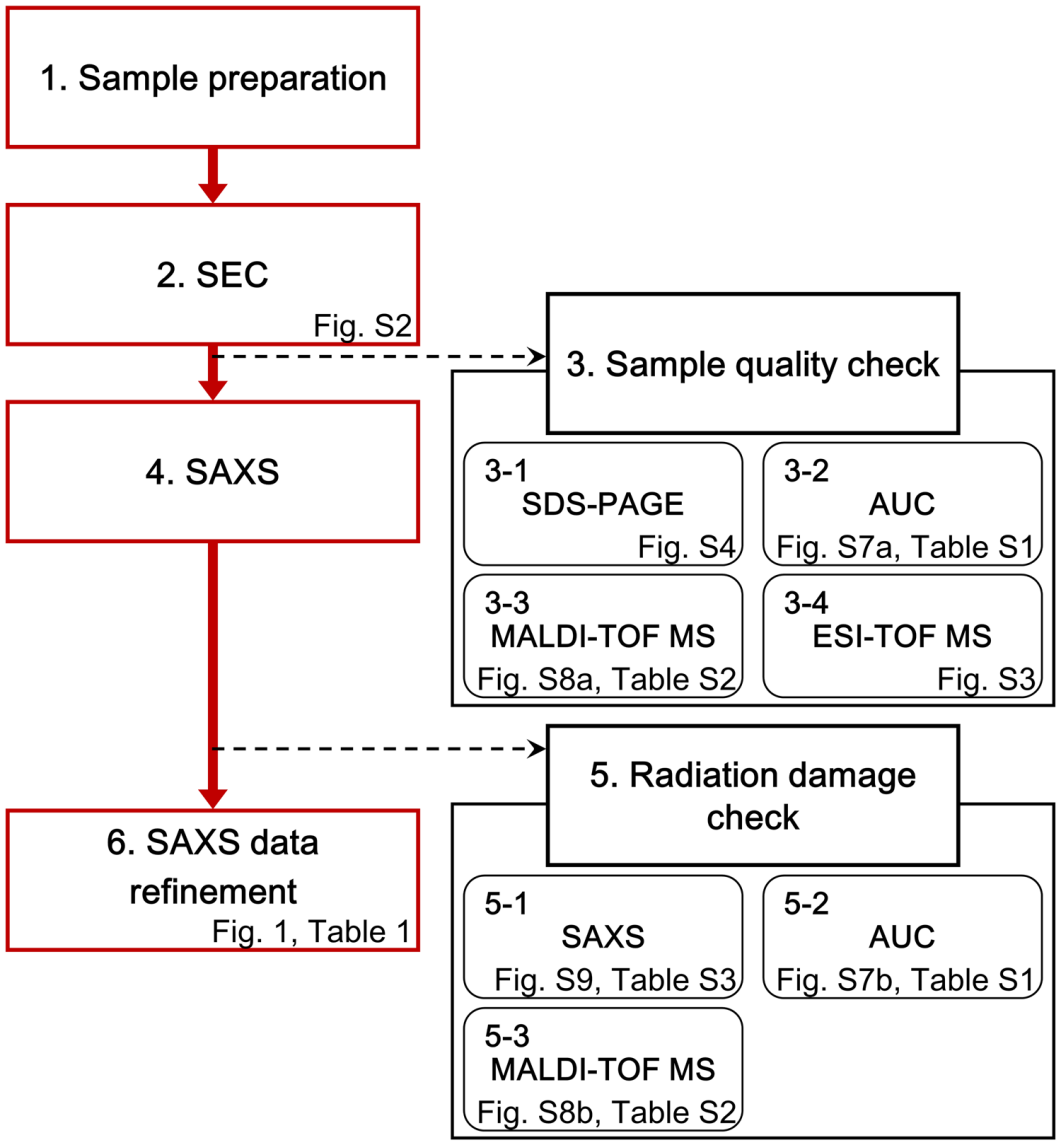
Flowchart of the work steps for aggregation-free SAXS measurement of ER-60. To obtain aggregation-free SAXS data, several methods were assembled. The procedure consists of sample preparation, purification with SEC, quality checks, SAXS measurement, radiation damage check, and data refinement.

We developed several methods to obtain aggregation-free SAXS data. Figure 8 shows the procedure, which consists of sample preparation, its purification (SEC), quality checks, SAXS measurement, radiation damage check, and data refinement. The entire procedure was performed as follows.

#### Sample preparation

Recombinant ER-60 was expressed as described previously^26^. Briefly, *E. coli* BL21(DE3) cells (Takara Bio, Inc., Shiga, Japan) transformed with expression plasmids encoding mature human ER-60 (Ser^25^-Leu^505^) without signal peptide were precultured in LB broth at 37 °C for 5 h. The expression was induced in the presence of 0.4 mM isopropyl-β-d-thiogalactopyranoside at 25 °C for 48 h. Purification procedures of the recombinant ER-60 were carried out at 4 °C. *E. coli* cells suspended in buffer A (20 mM HEPES buffer, pH 6.8, containing 50 mM KCl, 5 mM EDTA, and 1 mM phenylmethylsulphonyl fluoride) were disrupted by sonication and centrifuged at 10,000 × *g* at 4 °C. The expressed recombinant ER-60 was purified from the cell lysates using affinity column chromatography on AF heparin toyopearl 650 M (Tosoh, Tokyo, Japan) with buffer A at 4 °C. Recombinant ER-60 was eluted from the AF heparin column with 0.4 M KCl in buffer A and subsequently purified by ion-exchange column chromatography on Resource Q (GE Healthcare, IL, USA) with 20 mM Tris–HCl, pH 7.4 buffer containing 0.2 mM EDTA and 0.5 mM phenylmethylsulphonyl fluoride, followed by SEC on Superdex 200 Increase 10/300GL (GE Healthcare) with SEC buffer (20 mM Tris–HCl buffer, pH 7.4, containing 150 mM NaCl and 1 mM CaCl_2_) at 4 °C. A sample from each purification step was resolved by SDS-PAGE (Supplementary Fig. S4a–c).

To prepare oxidized or reduced ER-60, the purified samples were incubated in 0.1 mM GSSG or 10 mM DTT in SEC buffer for 2 h at 4 °C. Then, SDS-PAGE was performed to evaluate the oxidized or reduced state of the active centre cysteines in the ER-60 samples. The ER-60 solutions were incubated in 80 mM Tris/HCl buffer at pH 6.8, with or without 25 mM 4-acetamido-4-maleimidylstilbene-2,2-disulphonate (AMS) at 4 °C for 30 min. Then, they were diluted with 2 × Laemmli SDS sample buffer^27^ without reducing agent and incubated at 37 °C for 2 h. The samples were resolved by SDS-PAGE (Supplementary Fig. S4d). Free thiols of oxidized and reduced ER-60 were modified with three and seven AMS molecules, respectively, indicating that the dithiols of the active cysteine pair were fully oxidized and reduced as described previously^26^.

#### SEC procedure in SEC-SAXS

To reduce contamination and aggregates in the samples for SAXS measurements, SEC-SAXS was applied to the samples. In this measurement, a stopping mode in a laboratory-based SEC-SAXS system was adapted to increase the statistics of scattering intensity (see the “SAXS” section)^22^.

For SEC, Superdex 200 Increase 10/300GL was used as the separation column. The buffers that were used for the preparation of oxidized and reduced samples were flowed through the column at 25 °C. The initial injection volume and concentration of both samples were 500 μL and 5.0 mg mL^−1^, respectively, and the flow rate was 0.02 mL min^−1^. The eluted solution at the elution peak of the monomer was divided into two portions (shown in Table 1 and Supplementary Fig. S2). One portion was immediately provided for the SAXS measurement, and the other was used for its quality check.

#### Sample quality check

We examined the quality of the samples before the SAXS measurements.

##### SDS-PAGE

The first examination was SDS-PAGE. The samples were diluted with 2 × Laemmli SDS sample buffer with or without reducing agent (2-mercaptoethanol) and incubated at 37 °C for 2 h. The samples were resolved by SDS-PAGE (Supplementary Fig. S4e). There were no bands other than the main ones, demonstrating that the samples were neither truncated nor degraded.

##### Analytical ultracentrifugation 1

Next, AUC was conducted to determine the abundance of monomers, dimers, trimers, other aggregations, and contaminations in the sample solution. The AUC experiments were performed at 60,000 rpm at 25 °C with a sedimentation velocity method using Rayleigh interference optics (ProteomeLab XL-I, Beckman Coulter). The weight concentration distribution *c*(*s*_20,w_) as a function of the sedimentation coefficient was obtained by fitting the time evolution of sedimentation data with Lamm formula^28^ using SEDFIT software^29^. The sedimentation coefficient was also normalized to the value at 20 °C in pure water (*s*_20,w_). The molecular weight for each component was calculated using *s*_20,w_ and the friction ratio *f*/*f*_0_.

Supplementary Fig. S7a shows the weight concentration distribution *c*(*s*_20,w_). There was one major peak around *s* = 3.5, and two minor peaks around *s* = 5.2 and *s* = 7.0 in the AUC profiles of both oxidized and reduced samples. This means that the sample solutions were highly purified, but there were very small aggregates (*s* = 5.2 and *s* = 7.0). The detailed values are listed in Supplementary Table S1.

##### MALDI-TOF mass spectrometry 1

MALDI-TOF MS was used to confirm the aggregation numbers of the above-mentioned aggregates in the sample solutions. The samples and a saturated solution of sinapinic acid (Bruker Daltonics, Bremen, Germany) in TA30 (30% acetonitrile in 70% of 0.1% TFA in water) were mixed at a ratio 1:9 and dropped on the ground steel MALDI plate (Bruker Daltonics). After drying and crystallisation of the droplets, the measurement was conducted on a microflexLT MALDI-TOF mass spectrometer (Bruker Daltonics) in positive ion mode. External calibrations were performed using protein standards II. Mass spectra data were recorded with flexControl and analysed using FlexAnalysis (Bruker Daltonics).

Supplementary Fig. S8a shows the mass spectra, and details of the detected peaks are shown in Supplementary Table S2. There were three components in the oxidized and reduced sample solutions whose molecular weights corresponded well to the monomer, dimer, and trimer of ER-60. Therefore, the three components observed in the AUC measurements were identified as the monomer, dimer, and trimer in the order of the *s*-value.

##### LC-ESI-TOF mass spectrometry

As the final sample check, the average mass values of the samples were measured rigorously using LC-ESI-TOF mass spectrometry to determine whether ER-60 samples were truncated or degraded. One hundred-fifty mM ammonium acetate (pH 6.8) was flowed at 2 µL min^−1^ through a capillary column (0.3 mm, *φ* × 550 mm, Chemco Scientific, Osaka, Japan) packed with resin (TSK G3000 SW gel bed, Tosoh Corporation, Tokyo, Japan). Two microliters of ER-60 solutions were loaded into the column by a sample injector (Rheodyne, Cotati, CA, USA) with a 2 µL sample loop. The samples were directly analysed using a micrOTOF II mass spectrometer with an electrospray ionisation (ESI) source (Bruker Daltonics). The pressure of the nebuliser gas, temperature of the dry heater, flow of the dry gas, and capillary voltage were set at 0.5 bar, 250 °C, 3 L min^−1^, and 4000 V, respectively. The mass spectra were obtained in the positive ion mode with a scanning range of *m/z* 500–10,000. ESI-L Low Concentration Tuning Mix (Agilent Technologies, Santa Clara, USA) was used for internal calibration. Mass spectrum data were analysed using Bruker Compass Data Analysis 4.2.

Supplementary Fig. S3 shows the mass spectra, indicating that the measured average mass values of the oxidized and reduced samples (54,259.3 and 54,264.0, respectively) were in agreement with those calculated from their sequences (54,260.5 and 54,264.6, respectively). The values are summarized in Table 1. This clearly indicated that both samples were neither truncated nor degraded.

#### SAXS

SAXS measurements were performed using a NANOPIX (Rigaku, Tokyo, Japan). X-rays from a high-brilliance point-focused X-ray generator (MicroMAX-007HF, Rigaku, Tokyo, Japan) were focused with a confocal mirror (OptiSAXS) and collimated with the lower parasitic scattering slit system, “ClearPinhole”. The scattered X-rays were detected with a two-dimensional semiconductor detector (HyPix-6000, Rigaku, Tokyo, Japan) with a spatial resolution of 100 μm. Two sample-to-detector distance (SDD) conditions, 1330 mm and 300 mm, were used to cover a wide *q* range (0.01–0.70 Å^−1^), where, $q=(4\uppi /\lambda )\mathrm{sin}(\theta /2)$, with $\lambda (=1.54\mathrm{ \AA })$ and $\theta$ representing the X-ray wavelength and scattering angle, respectively.

In the lower-*q* range measurement (0.01–0.07 Å^−1^, SDD = 1330.0 mm), the SEC-SAXS measurement with “stopping mode”^22^ was employed to remove the unfavourable aggregates from the sample solution. Then, the scattering data were collected for 1120 min (10 min × 112). In the higher-*q* range measurement (0.04–0.70 Å^−1^, SDD = 300.0 mm), standard SAXS measurements were applied. The scattering data were collected for 500 min (10 min × 50).

The obtained two-dimensional scattering patterns were converted to one-dimensional scattering profiles by radial averaging. Subsequently, one-dimensional scattering profiles were corrected for the intensity of the incident beam and the transmission. Then, the scattering profile of a protein in solution was obtained by subtracting that of the buffer. Finally, the unit of the scatter intensity was converted to an absolute scale by comparison with the scattering intensity of water (*I*_water_ = 1.632 × 10^–2^ cm^−1^). All reductions were processed with SAngler^30^. The low-*q* and high-*q* profiles were connected between *q* = 0.061 Å^−1^ and 0.064 Å^−1^.

#### Radiation damage check

The SAXS measurements with the stopping and normal modes took a long time. Therefore, we examined the radiation damage on the samples with the SAXS data in the first and last 100 min and measured the amount of the remaining aggregates in the samples immediately after the SAXS measurements utilizing AUC and MALDI-TOF mass spectrometry.

##### SAXS profiles in the first and last 100 min

Supplementary Fig. S9 shows the Guinier plots of the time-averaged SAXS profiles of the oxidized and reduced samples in the first and last 100 min of the experiments. There was no deviation between the Guinier plots of the time-averaged SAXS profiles for both samples. In addition, there was no difference in the calculated *R*_g_ values, as listed in Supplementary Table S3. These values were different from those shown in Table 1 because these were calculated from SAXS profiles before AUC-SAS treatment. These data suggested that further aggregation could not occur during the SAXS measurements.

##### Analytical ultracentrifugation 2

Using the same procedure as Analytical ultracentrifugation 1, we identified the abundance of monomers, dimers, trimers, other aggregates, and contaminants in the sample solutions immediately after the SAXS measurements. Supplementary Fig. S7b shows the weight concentration distribution *c*(*s*_20,w_) after the SAXS measurement. The amount of aggregates did not change before and after SAXS, indicating that there were no further aggregates due to radiation damage. This is also clear from the peak positions and areas on *c*(*s*_20,w_) listed in Supplementary Table S1.

##### MALDI-TOF mass spectrometry 2

Using the same procedure as that used for MALDI-TOF MS 1, we confirmed the aggregation numbers of the above-mentioned aggregates in the sample solutions immediately after the SAXS measurements. Supplementary Fig. S9b also shows the mass spectra of the sample solutions immediately after the SAXS measurements. As expected, there were no changes in the peak positions, indicating that there were no additional aggregates except for those observed in the solution before the SAXS measurements.

#### SAXS data refinement

As indicated in Supplementary Table S1, whereas the major component (96 wt%) in both sample solutions was our targeting ER-60 monomer, there was still an unremovable small amount of aggregates (4%), mainly dimers and trimers. For data refinement, we conducted the AUC-SAS method to eliminate the effect of these aggregates and make the scattering data precise. AUC-SAS extracts the scattering profile of a target component from a polydispersed system^20^. Supplementary Fig. S10 shows the SAXS data refinement by the AUC-SAS method with the observed AUC data. The gyration radii were refined from 33.4 ± 0.2 Å and 32.0 ± 0.2 Å to 32.1 ± 0.2 Å and 30.8 ± 0.2 Å for oxidized and reduced ER-60, respectively.

Finally, we obtained aggregation-free SAXS profiles for detailed structural analysis.

### Coarse-grained molecular dynamics (CG-MD) simulation

We used the atomic-interaction-based coarse-grained model (AICG2+), which is the so-called “structure-based model” ^31^. In this model, each amino acid is represented by a single particle at the C_α_ atom position. The reference structure for the structure-based potential was prepared by replacing the mutated Ala^60^ of the crystal structure of ER-60 (3F8U) with Cys using the Phyre2 modelling server^17^. The C-terminal region of ER-60 was modelled using PyMOL^32^. In order to make the structural modulation, we also added both Debye-Hückel-type electrostatic interactions with an ionic strength of 150 mM and hydrophobic interactions to the standard AICG2+ potential^33^.

We regarded the three peptides as flexible loops: Gly^133^ and Leu^365^, which are located between the **a**–**b** and **b′**–**a′** domains, respectively, and the region from Gln^490^ to Leu^505^, which is a flexible C-terminal region (Supplementary Figs. S1 and S11). The flexible loop is determined based on following two points: (1) the reported crystal structures and solution NMR data (2ALB, 2DMM), most of inter-domain linkers are parts of folded regions. (2) The observed SAXS profiles can be reproduced without denaturing them from folded domains. First, we did not apply any structure-based potential for these loops to make them flexible. Second, we assigned + 1 charges to six Lys particles and − 1 charges to one Asp and three Glu particles in the flexible loops, respectively. Third, we treated the other regions as folded; the intra-domain native-contact potential was included. It should be noted that the **b**–**b′** domains were regarded as one domain. For these folded regions, the charge of each amino acid was determined based on the surface charges of the reference structure^34^.

We performed 40 individual Langevin dynamics simulations for 10^8^ MD steps at 300 K using the simulation software CafeMol^35^. The simulation snapshots were collected every 5000 MD steps and a total of 800,000 snapshots were recorded.

## Supplementary Information

Supplementary Information.

## Data Availability

The relevant data generated and analysed in this study are available from the corresponding authors upon appropriate request.

## Code availability

All software used in this study is publicly available at the URLs below. SAngler 2.0.0: http://pfwww.kek.jp/saxs/SAngler.html. SEDFIT 15.01c: http://www.analyticalultracentrifugation.com/sedfit.htm. Pepsi-SAXS-NMA: https://files.inria.fr/NanoDFiles/Website/Software/Pepsi-SAXS/MacOS/Pepsi-SAXS-NMA. GNOM: https://www.embl-hamburg.de/biosaxs/gnom.html. The Phyre2 modelling server: http://www.sbg.bio.ic.ac.uk/~phyre/. The PyMOL Molecular Graphics System, Version 1.8: https://pymol.org/2/. CafeMol: https://www.cafemol.org/. BBQ: http://biocomp.chem.uw.edu.pl/tools/bbq. SCWRL4: http://dunbrack.fccc.edu/SCWRL3.php/#availability.
