# Supplementary material for: Solution structure of multi-domain protein ER-60 studied by aggregation-free SAXS and coarse-grained-MD simulation
Source: Sci Rep. 2021 Mar 11;11:5655. doi: 10.1038/s41598-021-85219-0 (PMC7952739; doi:10.1038/s41598-021-85219-0)
Supplement: Supplementary file 1 — Supplementary Information. [file 41598_2021_85219_MOESM1_ESM.docx]

**SUPPLEMENTARY INFORMATION**

**Solution structure of multi-domain protein ER-60 studied by aggregation-free SAXS and coarse-grained-MD simulation**

Aya Okuda^ⱡ^, Masahiro Shimizu^ⱡ^, Ken Morishima, Rintaro Inoue, Nobuhiro Sato, Reiko Urade* and Masaaki Sugiyama*

Institute for Integrative Radiation and Nuclear Science, Kyoto University, Kumatori, Sennan-gun, Osaka 590-0494, Japan.

ⱡ: equal contribution

*: Correspondence e-mail: sugiyama@rri.kyoto-u.ac.jp, urade.reiko.8w@kyoto-u.ac.jp

**Supplementary figures and tables**

**
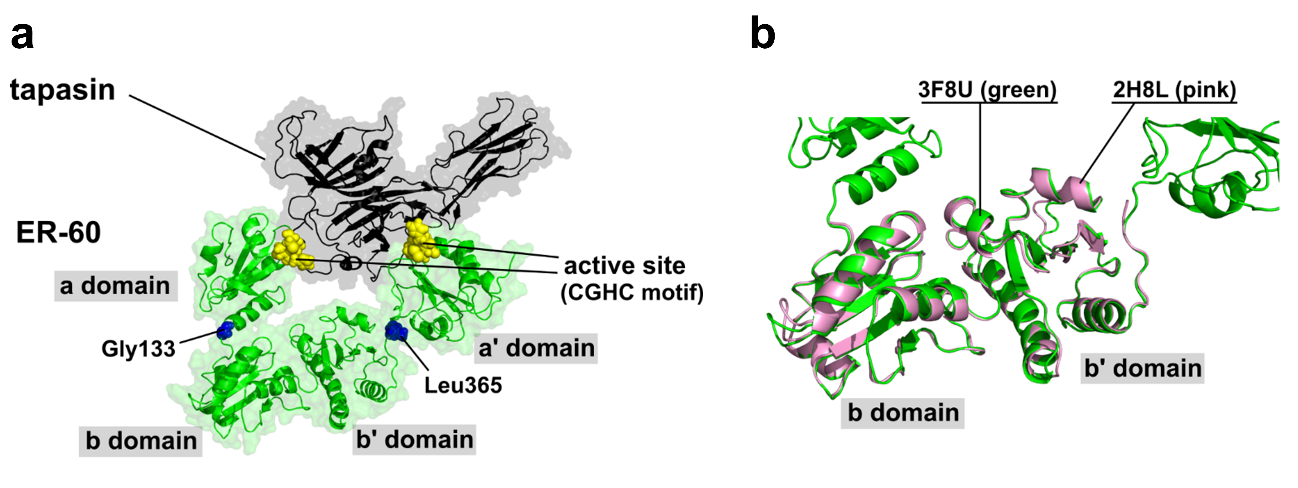
**

**Supplementary Fig. S1. Tertiary structure of ER-60.**

**a.** Crystal structure of full-length ER-60 with tapasin. Here, ER-60 is shown in green and tapasin in black. For ER-60, each domain is indicated and two active sites are shown as yellow spheres. In addition, Gly^133^ and Leu^365^ are shown as blue spheres, which were treated as flexible hinge residues in CG-MD simulation. In the crystal structure, an active site of **a** is mutated to CGHA, which is a mimic of the reduced state, and another active site of **a’** maintains CGHC as an oxidized state.

**b.** Uniformity of the **b**-**b’** domain structure. Here, two crystal structures (3F8U in green and 2H8L in pink) are aligned.

**
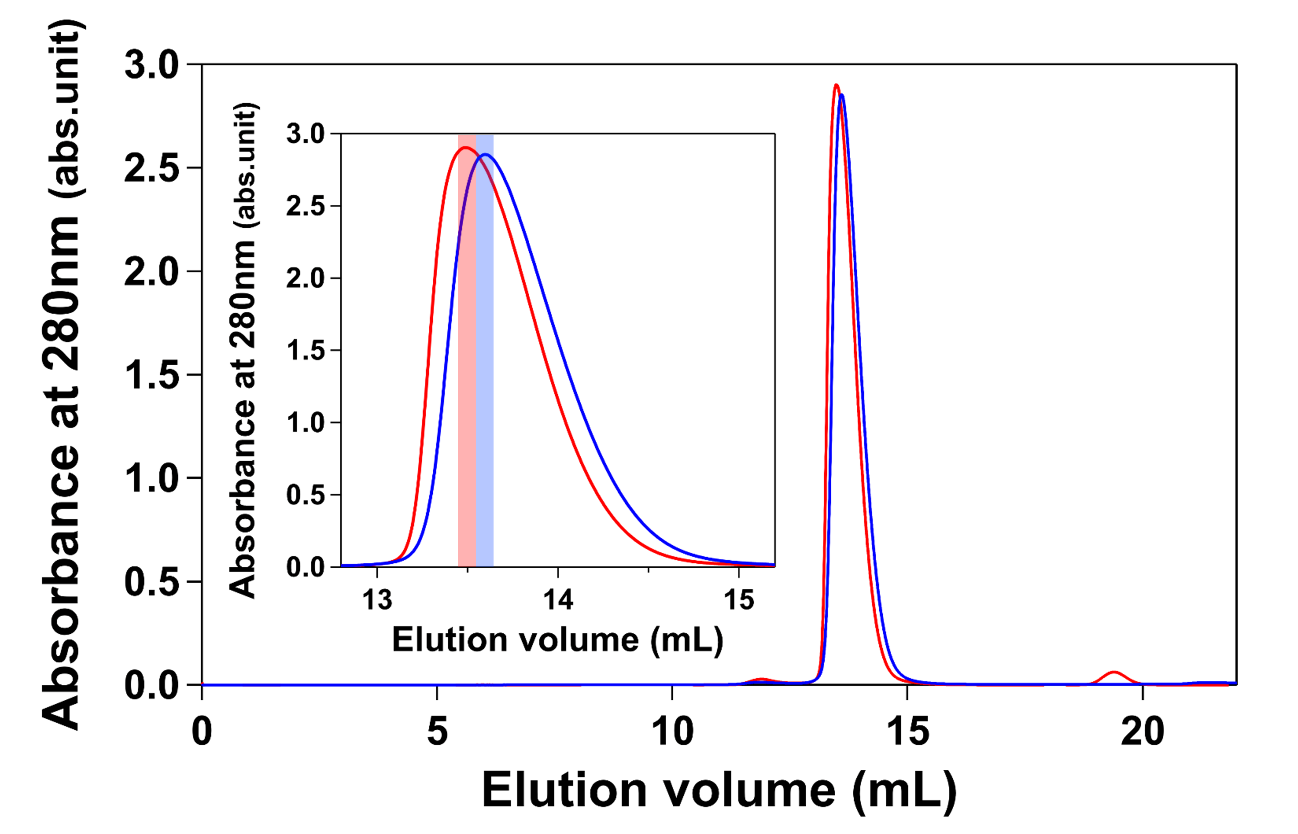
**

**Supplementary Fig. S2. SEC elution chart of ER-60 in SEC-SAXS.**

Red and blue lines indicate the elution charts of oxidized and reduced ER-60 in SEC-SAXS, respectively. The inset panel is the enlarged graphic in a range of 12.5–15.5 mL. Five hundred μL of each sample at 5.0 mg/mL concentration was loaded on the gel filtration column and eluted at the flow rate of 0.02 mL/min. Red and blue shade bars in the inset panel indicate the peak top fractions of oxidized and reduced ER-60, respectively.

**
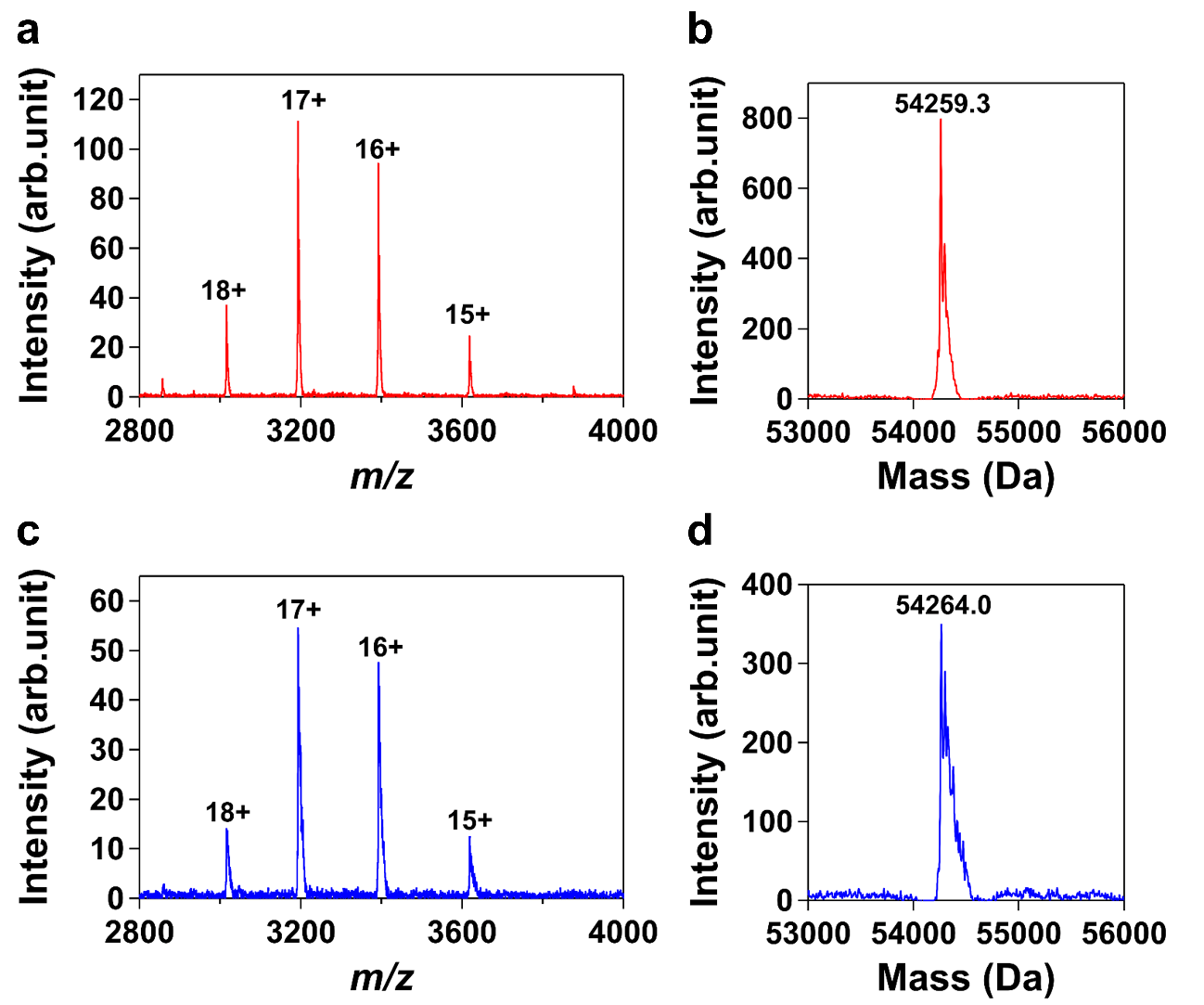
**

**Supplementary Fig. S3. LC-ESI-TOF Mass spectrum of ER-60.**

The left panels indicate LC-ESI-TOF mass spectrum of oxidized (red, **a**) and reduced (blue, **c**) ER-60. The right panels (**b, d**) indicate their deconvoluted mass spectrum.

**
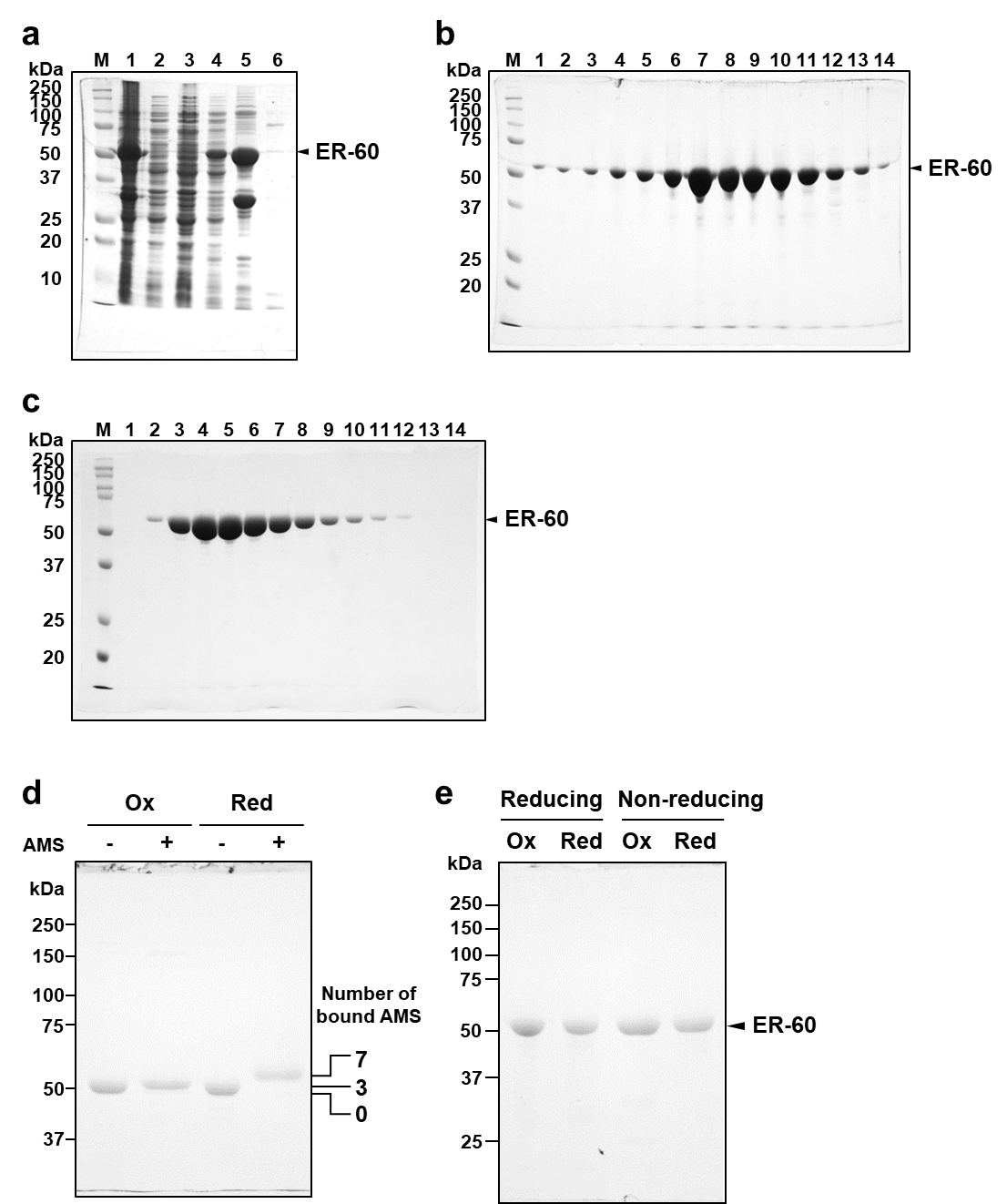
**

**Supplementary Fig. S4. SDS-PAGE of the ER-60 samples.**

**a. Purification of ER-60 by affinity column chromatography.** ER-60 expressed in the *E. coli* lysate (lane 1) was purified by affinity column chromatography on AF Heparin. Lane 2 indicates column through fraction. Lane 3 and lane 4 indicate column wash fraction with buffer A (20 mM HEPES buffer, pH 6.8, containing 50 mM KCl, 5 mM EDTA, and 1 mM phenylmethylsulphonyl fluoride). ER-60 was eluted from the column with 0.4 M (lane 5) and 0.75 M (lane 6) KCl in buffer A. Lane M indicates a molecular weight marker.

**b. Purification of ER-60 by anion exchange column chromatography.** ER-60, which was purified by affinity column chromatography, was further purified by anion exchange column chromatography on Resource Q. Lane M indicates a molecular weight marker. The fractions of lane 4-11 were subjected to the next purification step of SEC.

**c. Purification of ER-60 by SEC.** ER-60 was purified by SEC on Superdex 200 Increase 10/300GL finally. Lane M indicates a molecular weight marker. The fractions of lane 3-8 were subjected to SEC-SAXS.

**d. Thiol modification of oxidized (ox) and reduced (red) ER-60.** ER-60 was reduced and oxidized with DTT and GSSG, and either modified with AMS or not modified. Ox- and red-ER-60 treated with or without AMS were resolved by SDS-PAGE under non-reducing conditions.

**e. Quality check of ox- and red-ER-60 after SEC-SAXS.** Ox- and red-ER-60 were resolved by SDS-PAGE under reducing or non-reducing conditions. The gel was stained with Coomassie Brilliant Blue R-250.

**
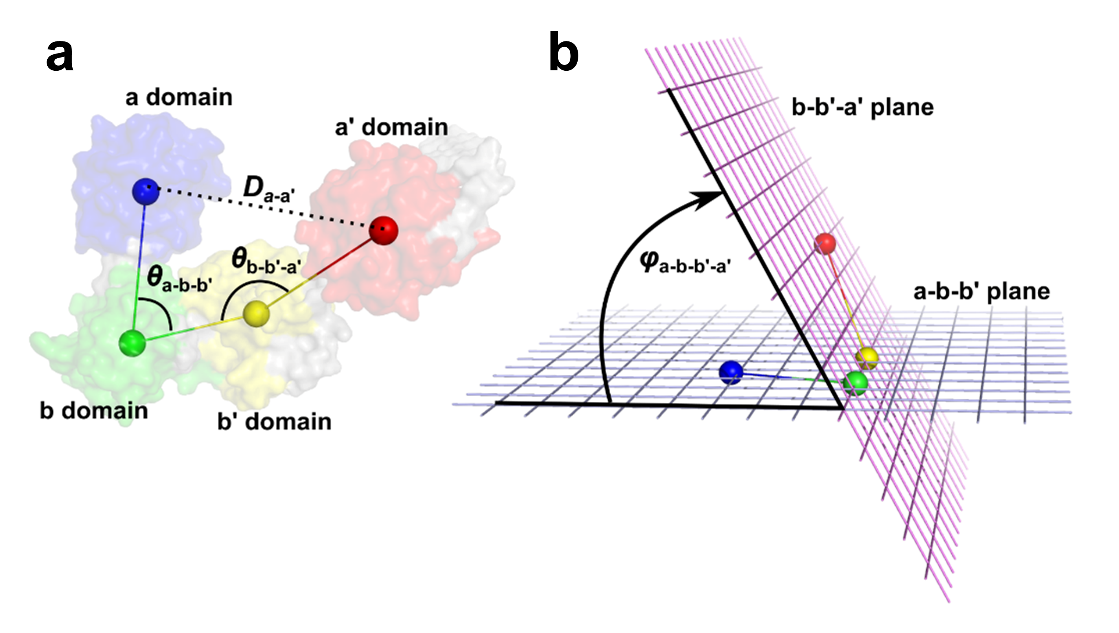
**

**Supplementary Fig. S5. Definition of the angles formed by the four domains.**

**a.** *θ***_a_**_-_**_b_**_-_**_b’_** and *θ***_b_**_-_**_b’_**_-_**_a’_** are angles formed by the **a**, **b**, **b’** and **b**, **b’**, **a’** domains, respectively. *D***_a_**_-_**_a’_** is the distance between the **a** and **a’** domains.

**b.** *φ***_a_**_-_**_b_**_-_**_b’_**_-_**_a’_** is defined as a dihedral angle by all four domains.

Here, the **a**, **b**, **b’**, **a’** domains and the inter-domain regions are coloured in blue, green, yellow, red, and gray, respectively.


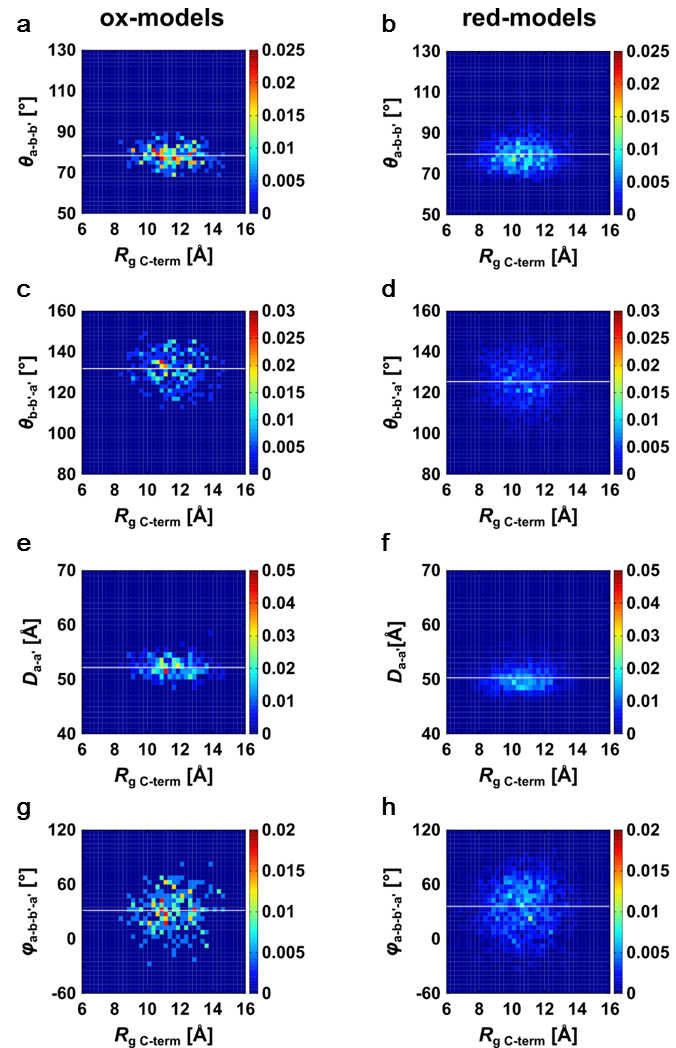


**Supplementary Fig. S6. Two-dimensional mapping of the conformation of C-terminal region and domain structure.**

Distributions of correlations between radius of gyration of C-terminal region (from Pro^488^ to Leu^505^) and *θ***_a-b-b’_** (**a, b**), *θ***_b-b’-a’_** (**c, d**), *D***_a-a_** (**e, f**), or *φ***_a-b-b’-a’_** (**g, h**) are shown for both ox- and red-models. Average of parameters are shown by white lines.

**
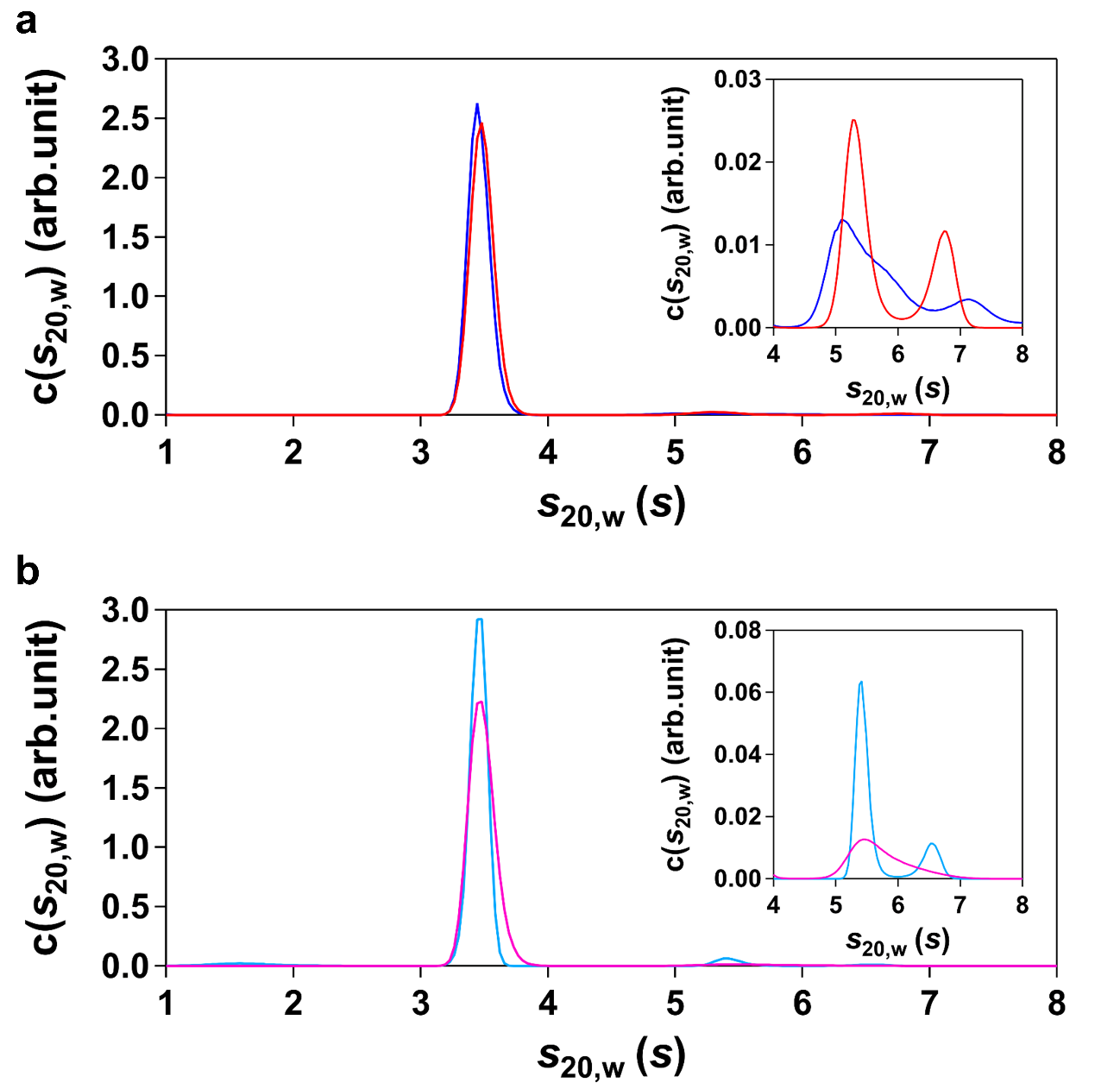
**

**Supplementary Fig. S7. Analytical ultra-centrifugation (AUC) profiles of ER-60.**

**a.** Red and blue lines indicate the AUC spectra of oxidized and reduced ER-60, respectively, before SAXS measurements.

**b.** Magenta and light blue lines indicate the AUC spectra of oxidized and reduced ER-60, respectively, after SAXS measurements.

Inset panels are enlarged pictures in an *S*_20,w_ range of 4–8 *s*.

**Supplementary Table S1. Values of ER-60 from the AUC profiles before and after SAXS measurement.**

|  |  | before SAXS measurement | | | | after SAXS measurement | | | |  |
| --- | --- | --- | --- | --- | --- | --- | --- | --- | --- | --- |
|  |  | *f*/*f*_0_ |  | *s*_20,w_ (s) | peak-area (%) | *f*/*f*_0_ |  | *s*_20,w_ (s) | peak-area (%) |  |
|  | oxidized | 1.49 | monomer | 3.5 | 97.0 | 1.48 | monomer | 3.5 | 97.9 |  |
|  |  |  | dimer | 5.3 | 2.0 |  | dimer | 5.4 | 1.1 |  |
|  |  |  | trimer | 6.8 | 1.0 |  | trimer | 5.9 | 1.0 |  |
|  | reduced | 1.50 | monomer | 3.5 | 97.1 | 1.48 | monomer | 3.5 | 96.2 |  |
|  |  |  | dimer | 5.1 | 2.4 |  | dimer | 5.4 | 3.1 |  |
|  |  |  | trimer | 7.1 | 0.5 |  | trimer | 6.5 | 0.7 |  |


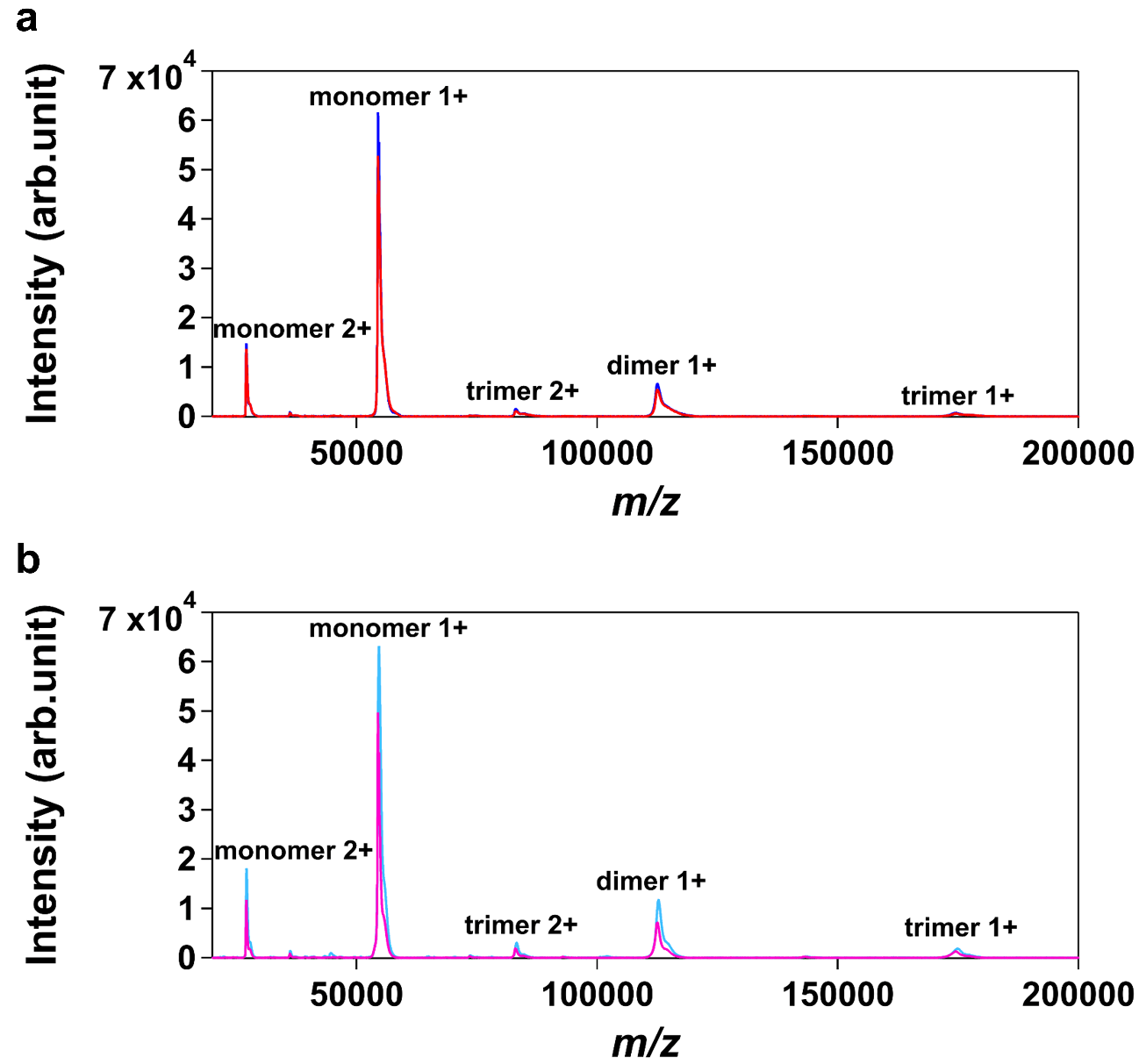


**Supplementary Fig. S8. MALDI-TOF Mass spectrum of ER-60.**

**a.** Red and blue lines indicate the mass spectra of oxidized and reduced ER-60, respectively, before SAXS measurements.

**b.** Magenta lines and light blue lines indicate the mass spectra of oxidized and reduced ER-60, respectively, after SAXS measurements.

**Supplementary Table S2. Peak information from the mass spectrum of ER-60.**

|  |  |  |  | before SAXS  measurement | |  | after  SAXS  measurement | |  |  |
| --- | --- | --- | --- | --- | --- | --- | --- | --- | --- | --- |
|  |  |  | charge state | *m/z* | Measured  average  mass (Da) |  | *m/z* | Measured  average  mass (Da) | Calculated  average  mass (Da) |  |
|  | oxidized | monomer | 2+ | 27066.399 | 54130.798 |  | 27071.585 | 54141.17 | 54260.5 |  |
|  |  | monomer | 1+ | 54447.899 | 54446.899 |  | 54430.636 | 54429.636 | 54260.5 |  |
|  |  | trimer | 2+ | 83119.42 | 166236.84 |  | 83014.788 | 166027.576 | 162781.5 |  |
|  |  | dimer | 1+ | 112518.151 | 112517.151 |  | 112508.172 | 112507.172 | 108521 |  |
|  |  | trimer | 1+ | 174258.065 | 174257.065 |  | 174298.294 | 174297.294 | 162781.5 |  |
|  | reduced | monomer | 2+ | 27074.618 | 54147.236 |  | 27145.513 | 54289.026 | 54264.6 |  |
|  |  | monomer | 1+ | 54445.125 | 54444.125 |  | 54568.848 | 54567.848 | 54264.6 |  |
|  |  | trimer | 2+ | 83130.365 | 166258.73 |  | 83208.509 | 166415.018 | 162793.8 |  |
|  |  | dimer | 1+ | 112522.451 | 112521.451 |  | 112776.738 | 112775.738 | 108529.2 |  |
|  |  | trimer | 1+ | 174477.647 | 174476.647 |  | 174778.188 | 174777.188 | 162793.8 |  |


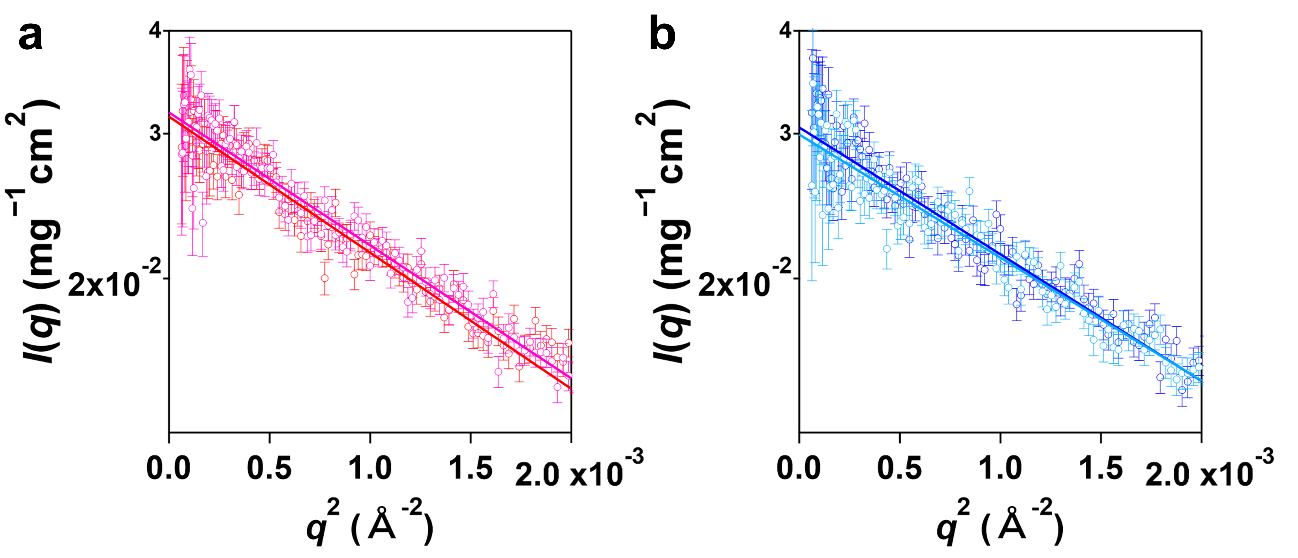


**Supplementary Fig. S9. Comparison of the Guinier plots of ER-60 at the initial and last 100 min of the experiments.**

**a**. Guinier plots from the SAXS profiles of oxidized ER-60. Red and magenta lines and circles indicate the averaged profiles of the first and last 100 min of the experiments, respectively.

**b.** Guinier plots from the SAXS profiles of reduced ER-60. Blue and light blue lines and circles indicate the averaged profiles of the first and last 100 min of the experiments, respectively.

**Supplementary Table S3. Calculated *R*_g_ values from the Guinier plots of ER-60 in the first and last 100 min of the experiments.**

|  |  |  | *R*_g_ (Å) |  |
| --- | --- | --- | --- | --- |
|  | oxidized | first 100 min | 33.8±0.5 |  |
|  |  | last 100 min | 33.4±0.5 |  |
|  | reduced | first 100 min | 32.6±0.5 |  |
|  |  | last 100 min | 32.1±0.5 |  |


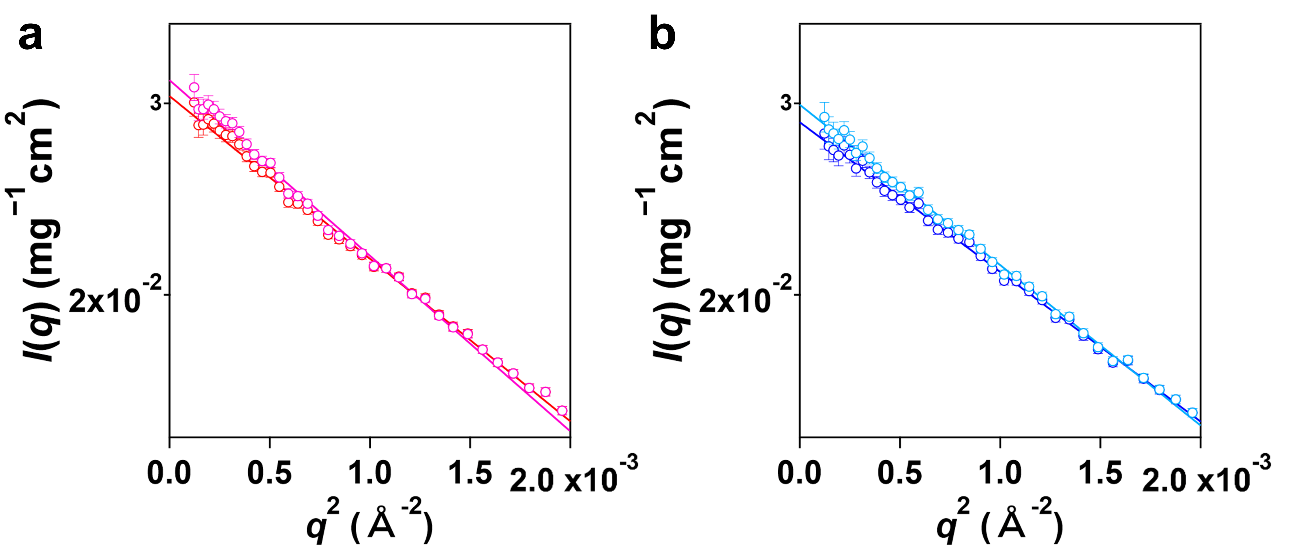


**Supplementary Fig. S10. Comparison of the Guinier plots of ER-60 before and after AUC-SAS treatment.**

**a**. Guinier plots from the SAXS profiles of oxidized ER-60. Magenta and red lines and circles indicate the profiles before and after AUC-SAS treatment, respectively.

**b.** Guinier plots from the SAXS profiles of reduced ER-60. Light blue and blue lines and circles indicate the profiles before and after AUC-SAS treatment, respectively.

**
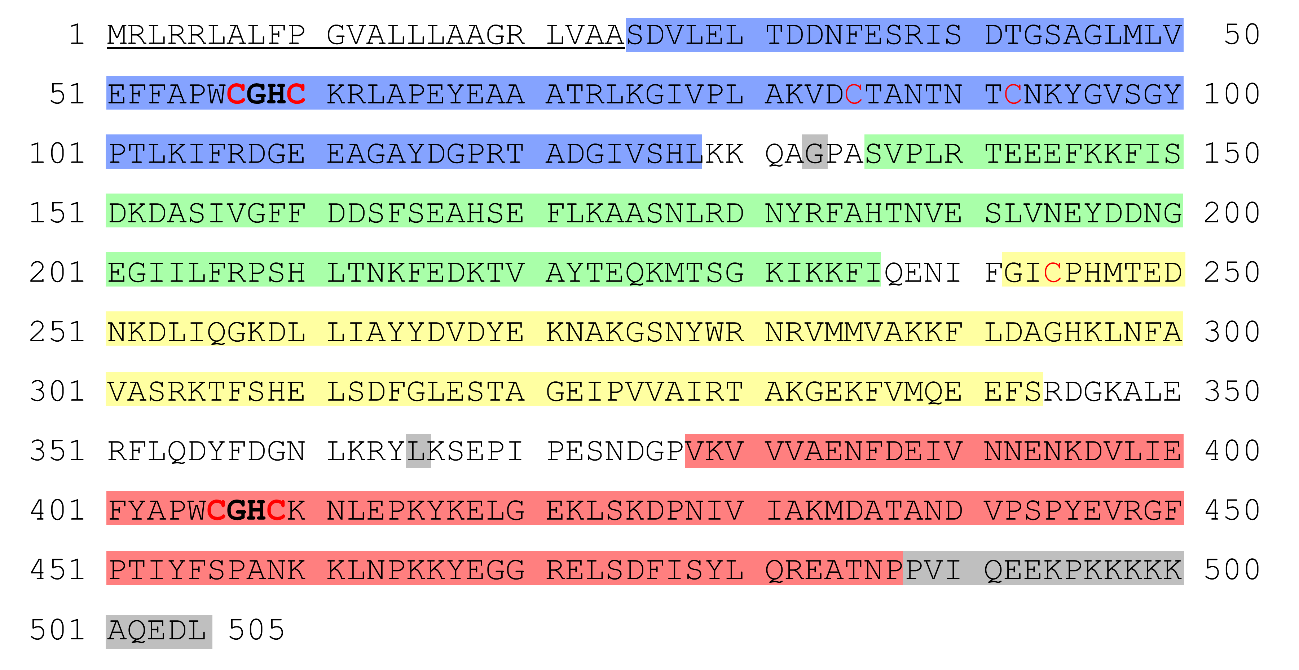
**

**Supplementary Fig. S11. Amino acid sequence of ER-60.**

The underline indicates signal peptides that were not included in recombinant ER-60. Blue, green, yellow, and red markers show the **a**, **b**, **b,** and **a’** domains, respectively. Gray markers exhibit the flexible regions set in the CG-MD simulation. Red and bold characters are cysteine and catalytically active CGHC motifs, respectively.

**Supplementary methods: Analysis of CG-MD trajectories**

**§1. Screening structural models that reproduce SAXS profiles**


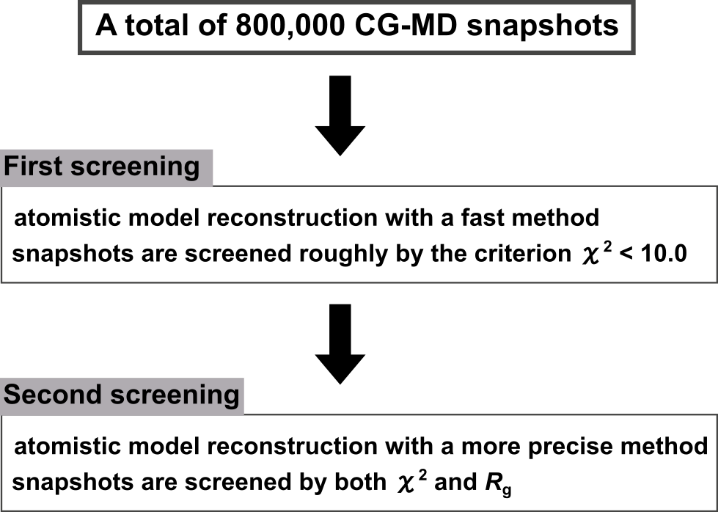
 We searched for structural models that reproduce aggregation-free SAXS profiles from simulation snapshots. A total of 800,000 snapshots were taken from CG-MD trajectories as structural models. Then, the SAXS profiles of the snapshots were calculated using the software Pepsi-SAXS^[[1]](#endnote-1)^ and were compared with the experimental profiles. Since Pepsi-SAXS requires an atomistic model as input data, the first step is to reconstruct atomistic models from the CG-MD snapshots. To reduce the computational cost of reconstructing numerous snapshots, we devised an efficient screening pipeline. The pipeline consists of the following two steps (Supplementary Fig. S12):

**Supplementary Fig. S12. Extracting structural models that reproduce SAXS profiles from CG-MD trajectories.**

**Step 1. Screening CG-MD simulation snapshots roughly with the *χ*^2^ value**

Step1-A: reconstruction of atomistic models with a fast-modelling method

First, most parts of ER-60 that can be regarded as rigid were transformed into atomistic models by superposing the crystal structure. The rigid regions are from Ser^25^ to Gln^131^, Ala^135^ to Arg^363^, and Ser^367^ to Pro^488^. Next, we reconstructed the atomistic structure for the other regions. We modelled the backbones and sidechains independently. The protein backbones were modelled using BBQ^[[2]](#endnote-2)^. The sidechains were reconstructed for each amino acid independently by superposing an atomistic amino-acid model on the backbone. The atomistic amino-acid models were extracted from the reference structure for CG-MD simulations (see the **CG-MD** section in the **Methods**). Superposition was performed using the N, C_α_, and C atoms of the backbone.

Step1-B: rough model-screening using the χ^2^ value

The SAXS profiles were calculated by Pepsi-SAXS and compared with experimental SAXS profiles using *χ*^2^. Snapshots that satisfied *χ*^2^ < 10.0 for *q* < 0.7 Å^−1^ were selected.

**Step 2. Searching for simulation snapshots that reproduce experimental SAXS profiles**

This process was performed for the snapshots that were selected after **Step 1**.

Step2-A: reconstruction of atomistic models with a more precise method

Atomistic models were re-reconstructed by BBQ and SCWRL4^[[3]](#endnote-3)^.

Step2-B: screening models with both the *χ*^2^ and *R*_g_ values

The SAXS profiles were calculated using Pepsi-SAXS. Using these values, we calculated *R*_g_ from the slope of the Guinier plot. Each profile was compared with the experimental profile of the oxidized or reduced ER-60. After comparison, the models were screened based on the criteria for both the *χ*^2^ and *R*_g_ values. The first criterion was *χ*^2^ < 7.0 for 0 < *q* < 0.7 Å^−1^. The second criterion was that the *R*_g_ value matches the experimental data. Here, we adopted models satisfying 31.9 Å < *R*_g_ < 32.1 Å or 30.6 Å < *R*_g_ < 31.0 Å for the oxidized and reduced ER-60, respectively.

**§2. Definition of the domain positions.**

In this study, we defined the positions of the **a**, **b**, **b’**, and **a’** domains as the geometric centre of the C_α_ atoms of the regions from Ser^25^ to Leu^128^, Ser^136^ to Ile^236^, Gly^242^ to Ser^343^, and Val^378^ to Pro^487^, respectively.

**Supplementary References**

1. Grudinin, S., Garkavenko, M. & Kazennov, A. *Pepsi-SAXS*: an adaptive method for rapid and accurate computation of small-angle X-ray profiles, *Acta Cryst.* **D73**, 449–464 (2017). [↑](#endnote-ref-1)
2. Gront, D., Kmiecik, S. & Kolinski, A. Backbone building from quadrilaterals: a fast and accurate algorithm for protein backbone reconstruction from alpha carbon coordinates. *J. Comput. Chem.* **28**, 1593–1597 (2007). [↑](#endnote-ref-2)
3. Krivov, G. G., Shapovalov, M. V. & Dunbrack, RL. Jr. Improved Prediction of Protein Side-Chain Conformations with SCWRL4. *Proteins: Struct., Funct., Genet.* **77**, 778–795 (2009). [↑](#endnote-ref-3)
